# Supplementary material for: A flexible ontology for inference of emergent whole cell function from relationships between subcellular processes
Source: Sci Rep. 2017 Dec 18;7:17689. doi: 10.1038/s41598-017-16627-4 (PMC5735158; doi:10.1038/s41598-017-16627-4)
Supplement: Supplementary file 1 — Supplementary information [file 41598_2017_16627_MOESM1_ESM.pdf]

Supplementary Information

A flexible ontology for inference of emergent whole cell function from relationships between subcellular processes

Jens Hansen[1,2], David Meretzky[1,2], Simeneh Woldesenbet[1,2,3], Gustavo Stolovitzky[4,5], and Ravi Iyengar[1,2]

[1] Department of Pharmacological Sciences and [2] Systems Biology Center New York, Icahn School of Medicine at Mount Sinai, New York NY 10029

[3]Department of Life Science, IMC University of Applied Sciences Krems, Kremsan der Donau, Austria

[4]Thomas J. Watson Research Center, IBM, Yorktown Heights, NY USA and [5]Department of Genetics and Genomics Sciences, Icahn School of Medicine at Mount Sinai, New York NY

Address Correspondence to

Ravi Iyengar

Department of Pharmacological Sciences

Icahn School of Medicine at Mount Sinai

1425 Madison Room 12-70

New York NY 10029

Phone: 212-659-1707

e-mail Ravi.Iyengar@mssm.edu

Supplementary Figure S1

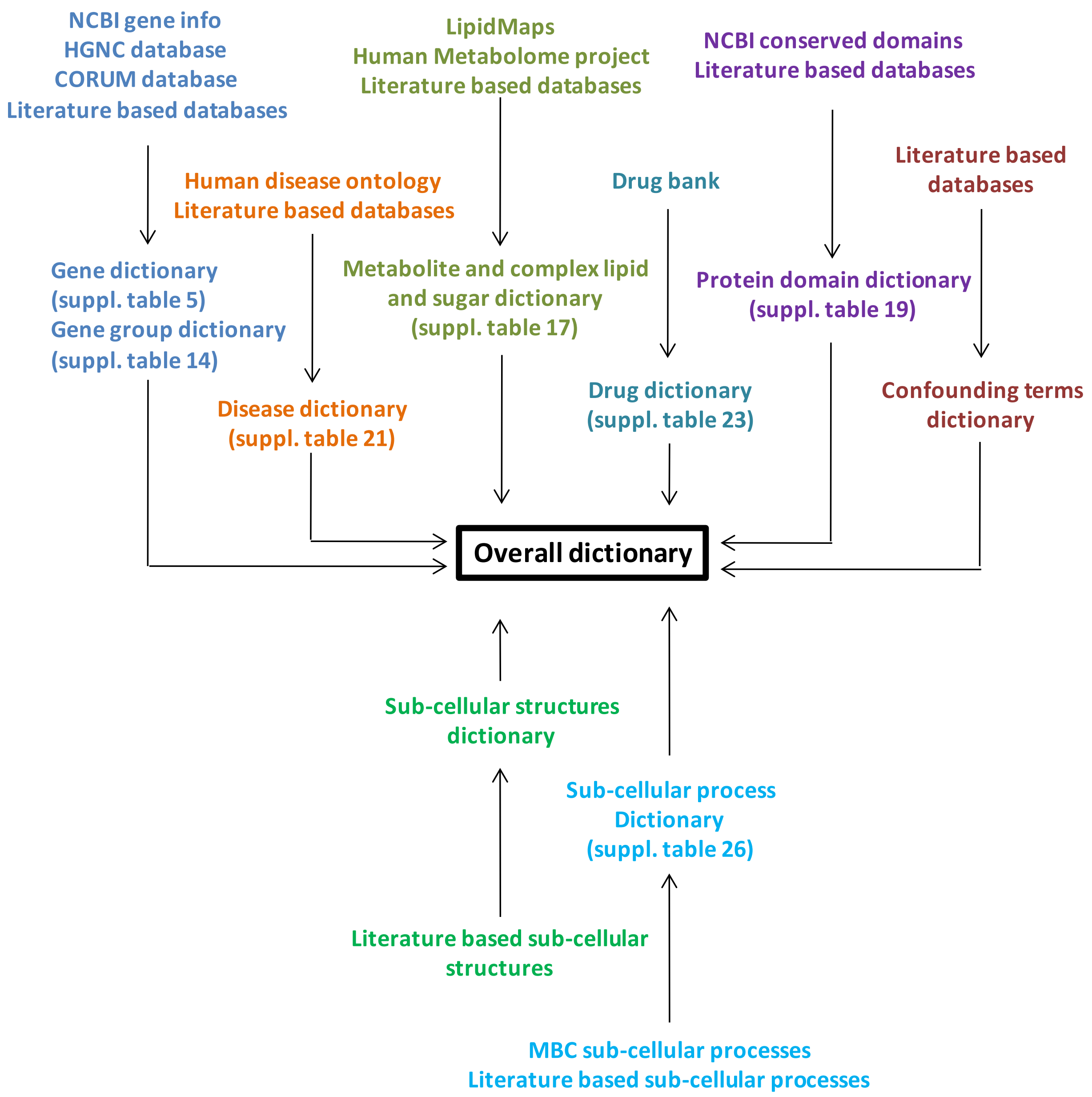

Supplementary Fig. S1: Different dictionaries that were merged to generate the final dictionary and the databases that were used to generate them. Different colors indicate different biological entity classes. Also shown are the Gene dictionary and the Gene group dictionary belonging to the biological entity class 'Genes and proteins', all other dictionaries refer to that biological entity class that is indicated in their names.

Supplementary Figure S2

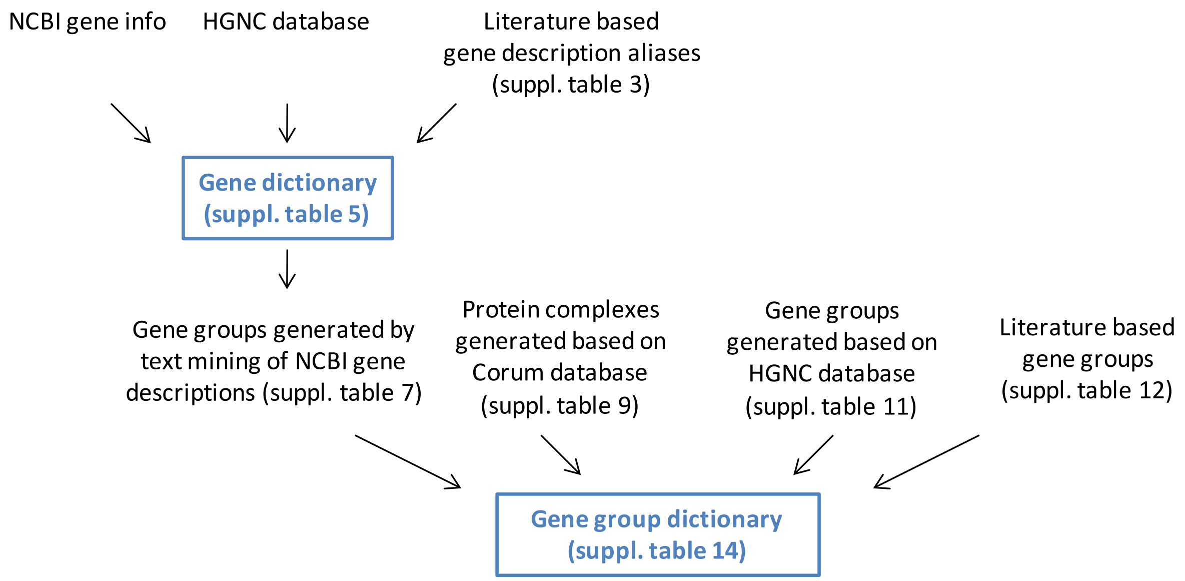

Supplementary Fig. S2: Generation of the Gene dictionary and the Gene group dictionary.

Supplementary Figure S3

Gene dictionary

NCBI gene info

HGNC database

Literature based gene description aliases and synonyms (suppl. table 3)

Combine databases

Remove synonyms that are only numbers
(e.g. the synonym '464.2' for the NCBI symbol 'CCL3L1')

Search for descriptions that contain terms in brackets
(e.g. 'ATP-binding cassette, sub-family F (GCN20), member 2')

a) add terms in brackets as alias description after manual validation (suppl. table 4)
(add 'GCN20')

b) replace terms in front of brackets by terms in brackets and add as alias descriptions
(add 'GCN20, member 2')

c) remove terms in brackets and add as alias descriptions
(add 'ATP-binding cassette, sub-family F, member 2')

Search for descriptions and alias descriptions that contain words starting with 'N-' (nitrogen) or 'O-' (oxygen) and generate a description alias by removing these letters
('procollagen N-endopeptidase' → 'procollagen endopeptidase')

Search for descriptions and alias descriptions that contain the words 'protease' or 'peptidase' and generate a description alias by replacing 'protease' with 'peptidase' or 'peptidase' with 'protease'
(e.g. 'apoptotic protease activating factor 1' → 'apoptotic protease activating factor 1')

Search for final descriptions that contain 'cytochrome P450' and replace 'cytochrome P450' plus the number that follows "CYP" in the NCBI symbol
(e.g. 'cytochrome p450, family 11, subfamily a, polypeptide 1' (NCBI symbol 'CYP11A1') → 'cytochrome P450 11A1')

Gene dictionary
(suppl. table 5)

Supplementary Fig. S3: Generation of the Gene dictionary.

Supplementary Figure S4A

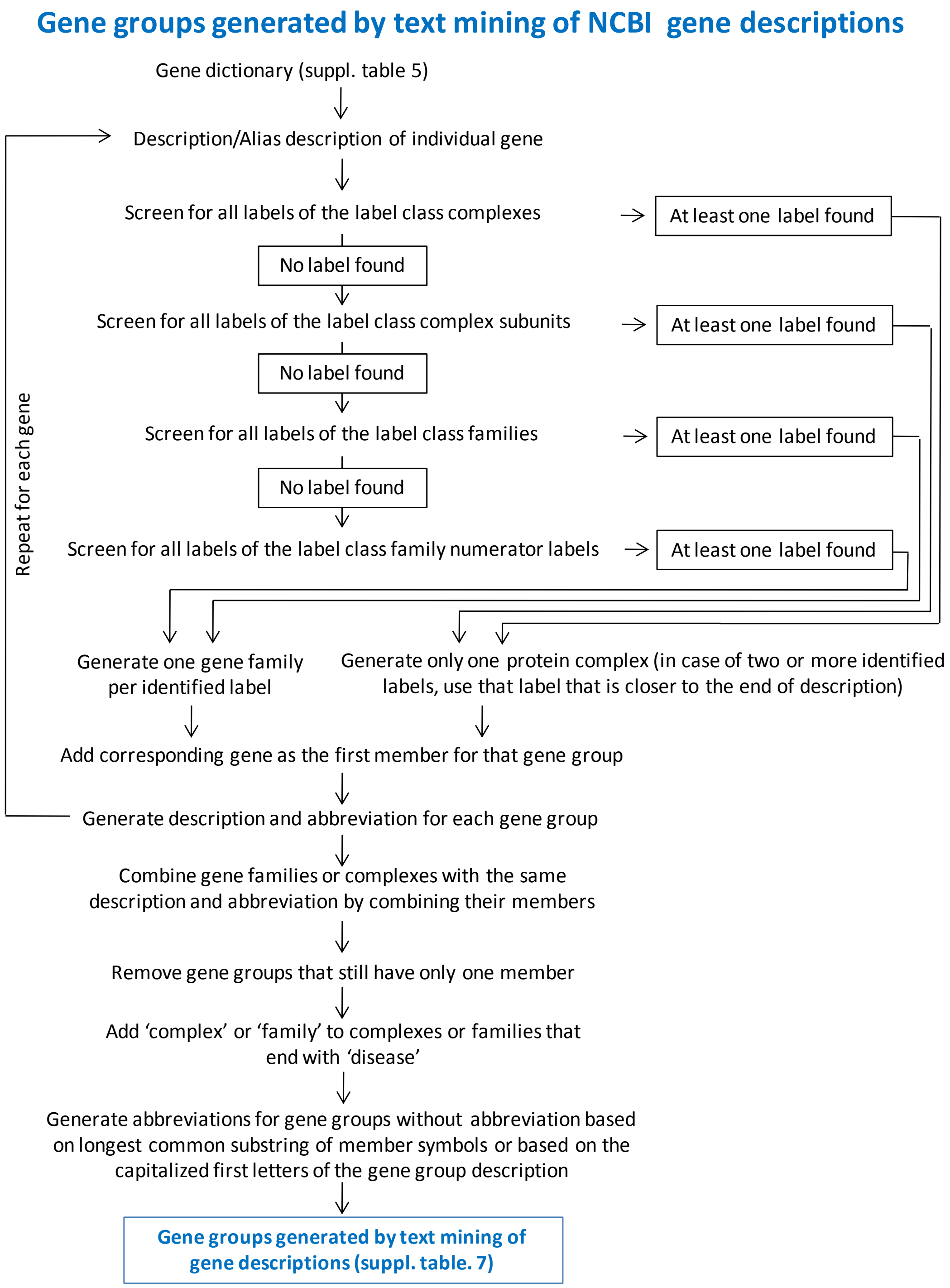

Supplementary Fig. S4A: Generation of protein complexes and gene families via a text mining based approach. Descriptions and description aliases of all genes in the gene dictionary were screened to predict protein complexes and gene families.

Supplementary Figure S4B

Gene groups generated by text mining of NCBI gene descriptions

Full gene name with identified complex label

(that is not followed by any gene group exclusion word [suppl. table 6])

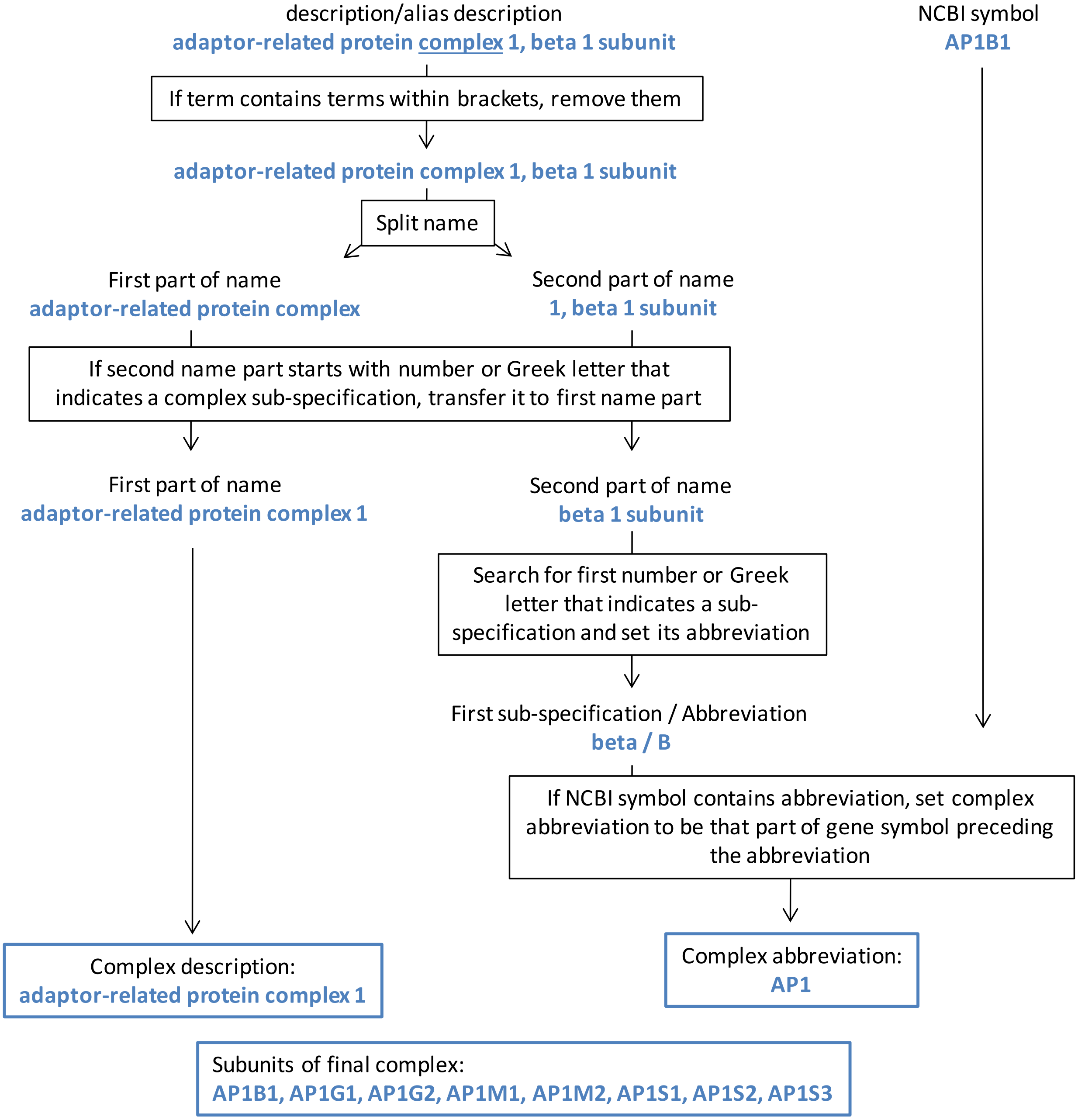

Supplementary Fig. S4B: Generation of descriptions and abbreviations for complexes that were identified based on the label 'complex'.

Supplementary Figure. S4C

Gene groups generated by text mining of gene descriptions

Full gene name with identified complex subunit or family label

(that is not followed by any gene group exclusion word [suppl. table 6])

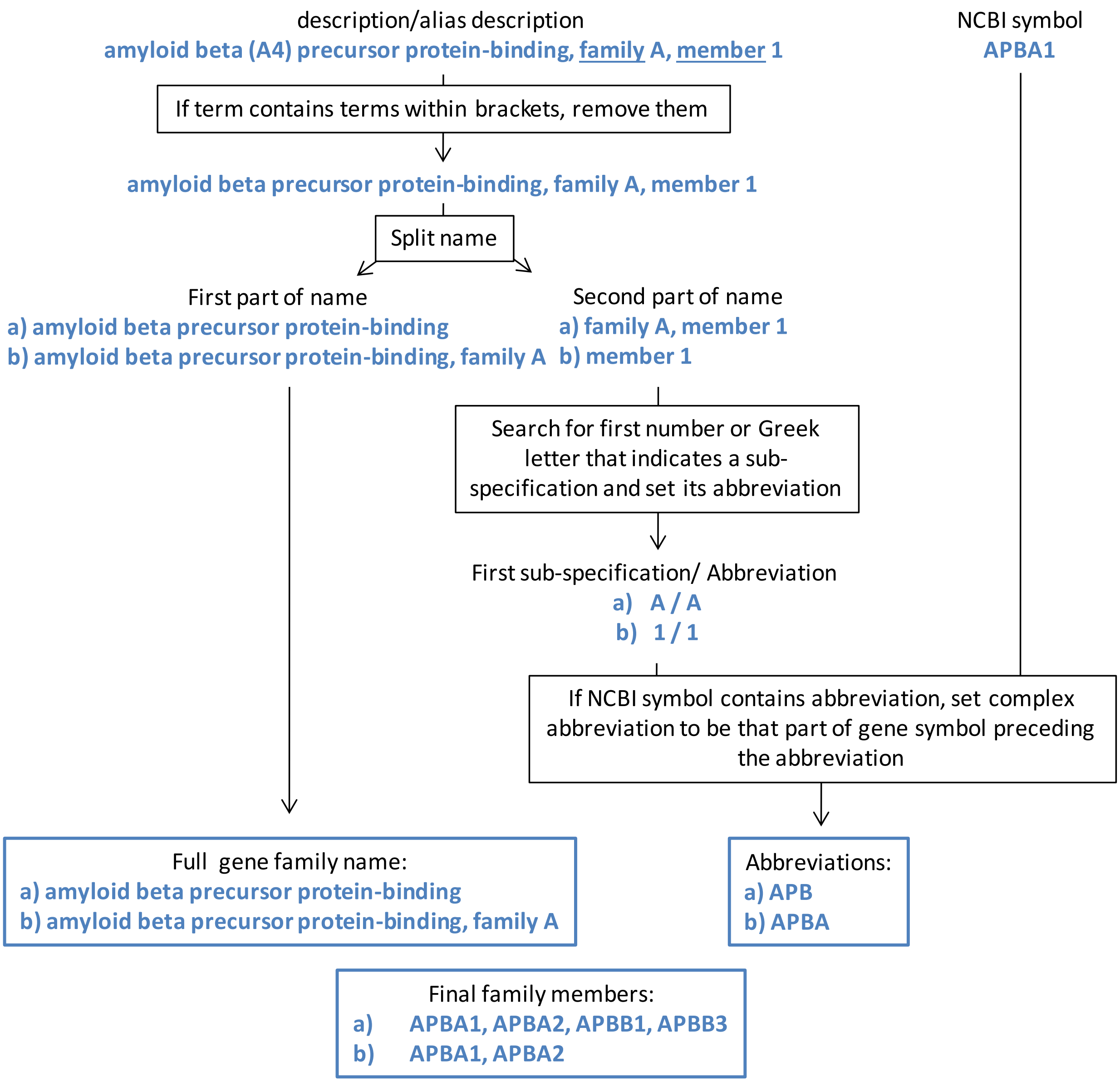

Supplementary Fig. S4C: Generation of descriptions and abbreviations for complexes and families that were identified based labels of the complex subunit set or family set.

Supplementary Figure. S4D

Gene groups generated by text mining of NCBI gene descriptions

Full gene name with identified sub-descriptor label

(that is not followed by any gene group exclusion word [suppl. table 6])

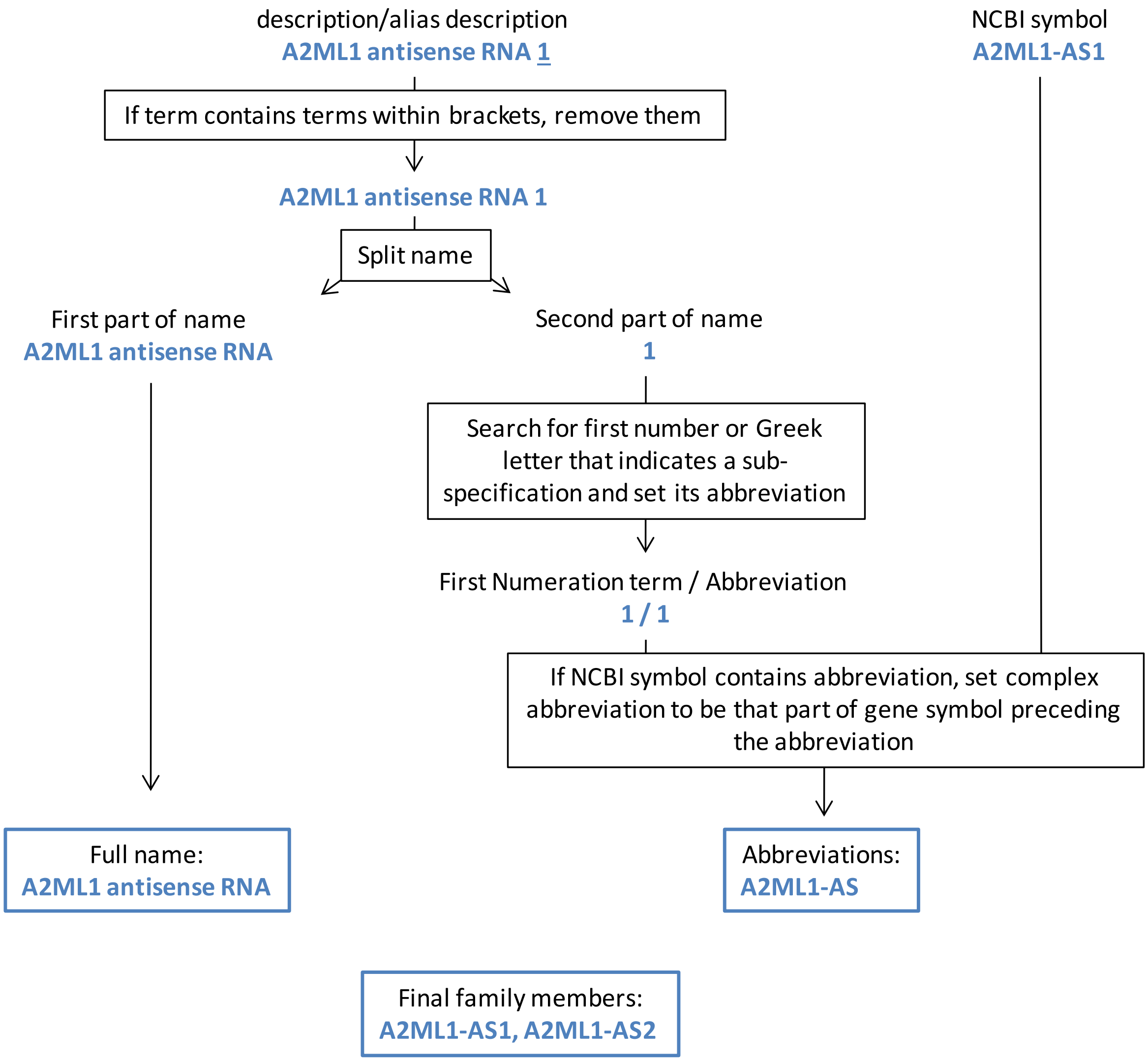

Supplementary Fig. S4D: Generation of descriptions and abbreviations for families that were identified based labels of the family enumerator set.

Supplementary Figure S4E

Gene groups generated by text mining of NCBI gene descriptions

If an abbreviation could not be generated as described in suppl. figures 4b, 4c or 4d,
set abbreviation to be the longest shared substring of all its member symbols:
e.g. :
Complex full name: **ATP synthase H+ transporting mitochondrial Fo**
Complex abbreviation: **ATP5**
Complex subunits: **ATP5F1,ATP5G1, ATP5G2, ATP5G3, ATP5H, ATP5I, ATP5J, ATP5J2, ATP5L, ATP5L2**

Supplementary Fig. S4E: Alternative approach for the generation of gene group abbreviations. If an abbreviation could not be generated as described in Supplementary Fig. S4B, S4C or S4D, the abbreviation was set to be the longest shared substring of all its member symbols, if this consisted of at least 3 letters. Otherwise we used the capitalized first letters of the description.

Supplementary Figure S4F

Complexes generated based on CORUM database

CORUM complex database

↓

Keep only human complexes

↓

Add NCBI symbols of complex subunits based on entrez IDs

↓

Remove any complexes with only one subunit

↓

Remove all complexes that contain two or more NCBI symbols or synonyms in their description
(e.g. ‘BCL6-HDAC4 complex’)

↓

Remove any terms following the first dot, semicolon or comma from description
and add as new description alias
(e.g. description: ‘Succinyl-CoA synthetase, GDP-forming’
description alias: ‘Succinyl-CoA synthetase’)

↓

Remove any terms in brackets from description and add as new description alias
(e.g. description: ‘BLOC-3 (biogenesis of lysosome-related organelles complex 3)’
description alias: ‘BLOC-3’)

↓

If first word of description is not a number followed by S, set this word as complex abbreviation
(e.g. description: ‘BLOC-3 (biogenesis of lysosome-related organelles complex 3)’
abbreviation: ‘BLOC-3’
number followed by S: ’20S proteasome’)

↓

Replace selected complex abbreviations and add manually generated description aliases
(suppl. table 8)

↓

Complexes generated based on CORUM database (suppl. table 9)

Supplementary Fig. S4F: Generation of complexes based on the CORUM database.

Supplementary Figure S4G

Gene groups generated based on HGNC database

HGNC database

↓

Remove plural 's' from any family description

↓

Search for the longest shared substring between any member symbols
If this consists of at least 3 letters, set abbreviation as this substring
(description: 1-acylglycerol-3-phosphate O-acyltransferase
symbols: 'AGPAT1', 'AGPAT2', 'AGPAT3', 'AGPAT4', 'AGPAT5',
'GPAT3', 'GPAT4', 'LCLAT1' ,'LPCAT1', 'LPCAT3' ,'LPCAT4',
Abbreviation: 'APGAT')

↓

Search for any descriptions that contain terms in brackets
(description: 5-hydroxytryptamine (serotonin) receptor, G protein-coupled)
a) Add terms in brackets of description as description aliases
(new description alias: serotonin receptor , G protein-coupled)
b) Add description without terms in brackets as description aliases
(new description alias: 5-hydroxytryptamine receptor, G protein-coupled)

↓

Define any families that contain one of the labels 'complex', 'subunit', 'signalosome' or 'proteasome' to be complexes
(e.g. 'Actin related protein 2/3 complex')

↓

Add literature based abbreviations as abbreviation aliases
(suppl. table 10)

↓

Gene groups generated based on HGNC database (suppl. table 11)

Supplementary Fig. S4G: Generation of gene families based on the HGNC database.

Supplementary Figure S4H

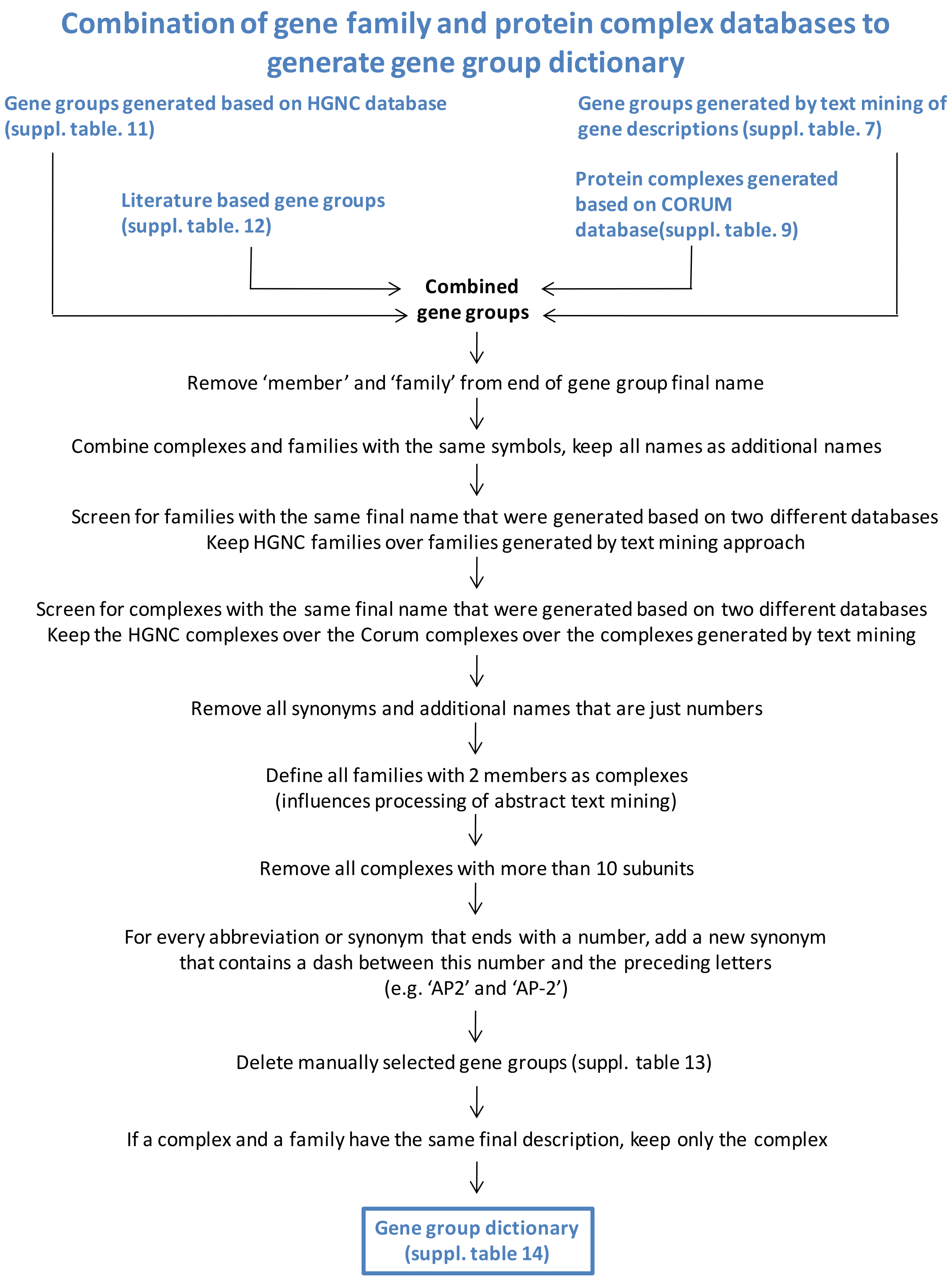

Supplementary Fig. S4H: Generation of the final gene group dictionary.

Supplementary Figure S5

Metabolites and complex lipid and sugar dictionary

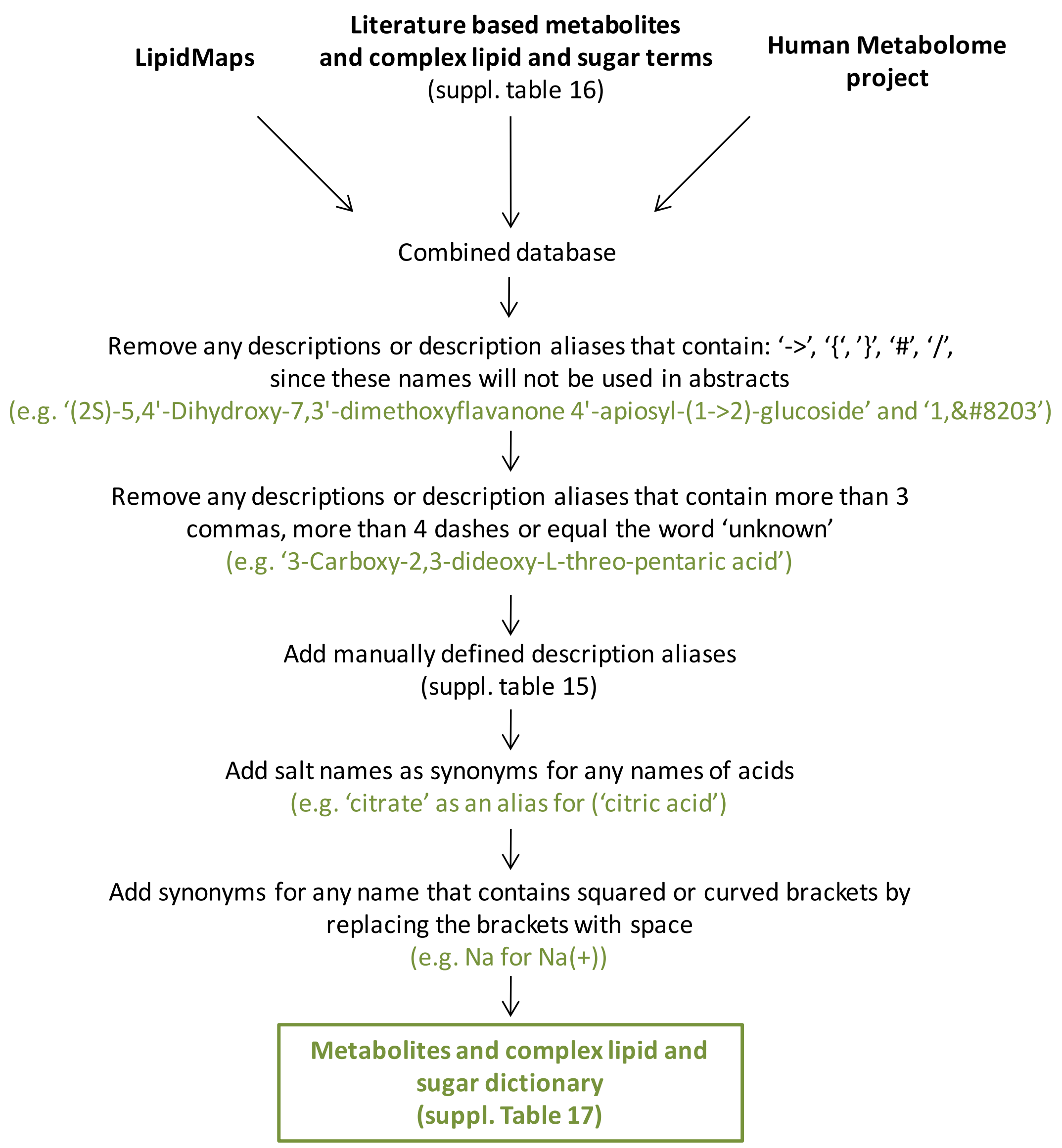

Supplementary Fig. S5: Generation of the Metabolites and complex lipids and sugars dictionary.

Supplementary Figure S6

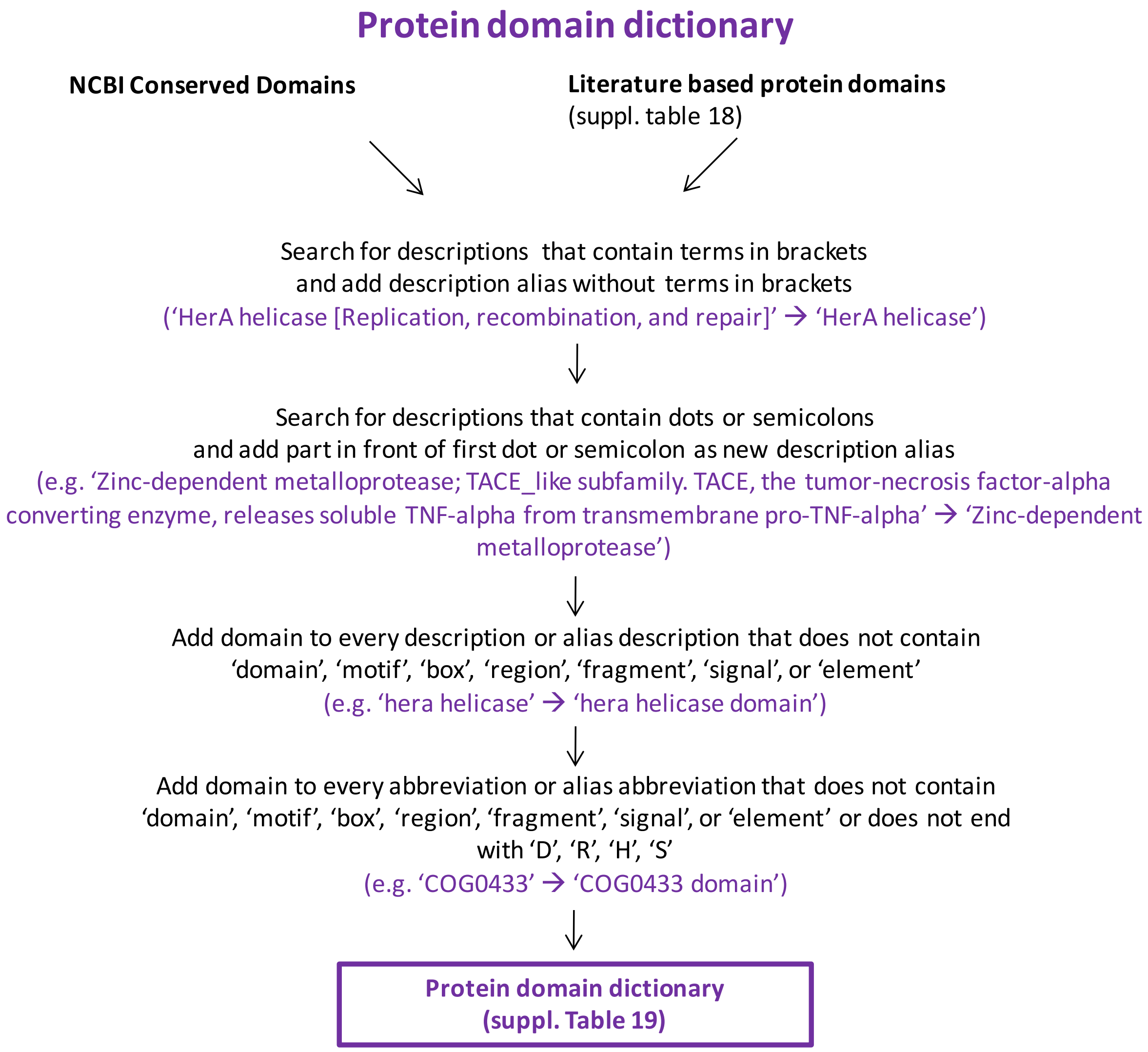

Supplementary Fig. S6: Generation of the Protein Domains and sugars dictionary.

Supplementary Figure S7

Disease dictionary

Human disease ontology

Literature based diseases (suppl. Table 20)

↓

Consider only descriptions that do not start with 'disease' or 'syndrome'
(these are parent nodes in the ontology that do not specify diseases any more)

↓

If a description contains apostrophe s, generate alias description without apostrophe s
(e.g. 'active cochlear Meniere's disease' → 'active cochlear Meniere disease')

↓

If description contains the term 'disease' generate alias description without term 'disease'
(e.g. 'active cochlear Meniere disease' → 'active cochlear Meniere')

↓

Set abbreviation to be the capitalized first letters of description, if at least 3 letters
(e.g. 'active cochlear Meniere disease' → 'ACMD')

↓

Disease dictionary (suppl. table 21)

Supplementary Fig. S7: Generation of the Disease dictionary.

Supplementary Figure S8

Drug dictionary

Drug bank database

Literature based drugs (suppl. table 22)

↓

Drug dictionary (suppl. table 23)

Supplementary Fig. S8: Generation of the Drug dictionary.

Supplementary Fig. S9

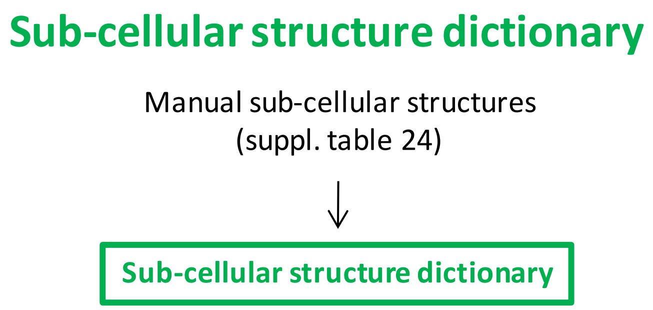

Supplementary Fig. S9: Generation of the Sub-cellular structure dictionary.

Supplementary Fig. S10

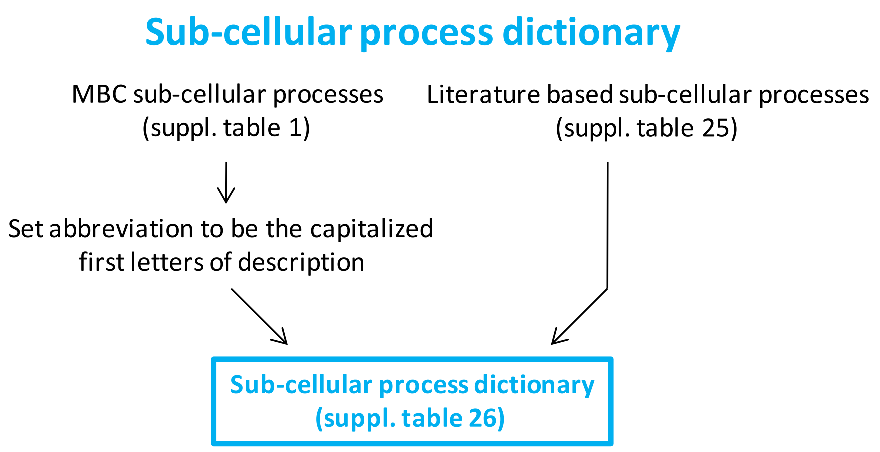

Supplementary Fig. S10: Generation of the Sub-cellular process dictionary.

Supplementary Fig. S11

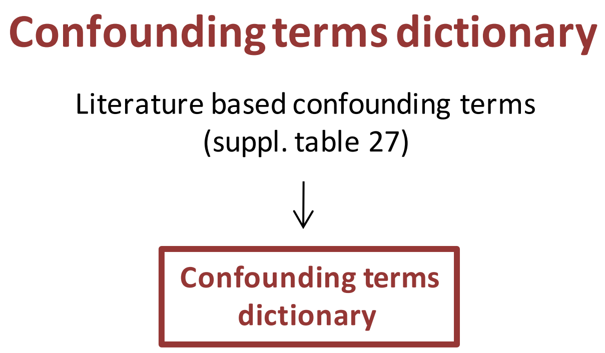

Supplementary Fig. S11: Generation of the Confounding terms dictionary.

Supplementary Figure S12

Combined dictionaries

↓

For any keys that start with 'eukaryotic' generate new key without 'eukaryotic'
(e.g. 'eukaryotic translation initiation factor 1' → 'translation initiation factor 1 ')

↓

Remove keys that are part of background words
(suppl. table 28)

↓

Remove keys that are numbers or one letter followed by numbers
(e.g. '1-4', 'C-1', 'M14')

↓

Remove keys with less than 3 characters

↓

Remove keys that contain the character '%'

↓

Remove keys that are combinations of three capital base pair letters
(e.g. 'ATC')

↓

For any key that ends with space followed by a number add a new key after replacing space by dash
(e.g. '1-AGPAT 6' → '1-AGPAT-6')

↓

Tokenize keys and recombine the words to one final expression
that is compatible with our abstract text mining algorithm

↓

Overall dictionary
(suppl. table 29)

Supplementary Fig. S12: Generation of the overall dictionary after merging of the individual dictionaries as shown in Supplementary Fig. S1.

Supplementary Figure S13

Tokenization flow chart

Title followed by abstract

↓

Split into text blocks based on delimiters
(‘{‘, ’/’, ‘\’, ‘(‘, ‘{‘, ‘,’, ‘.’, ‘?’,’,’!’,’}’,’)’,’@’,’;’,’:’,’}’)
Keep delimiter at end of each text block

↓

Replace squared or curly brackets by round brackets

↓

Recombine adjacent text blocks, if bracket(s) was(were) within a word
(‘PtdIns(‘ + ‘4,’ + ‘5)’ + ‘P2’ is recombined to ‘PtdIns(4,5)P2’)

↓

Recombine adjacent text blocks, if the previous text block does not end with the delimiters ‘/’ or ‘\’ and the text block does not start with space
(‘2.’ + ‘9’ is recombined to ‘2.9’)

↓

Remove delimiters from end of text block and add as own text block
(1 text block: ‘Role of plasma-membrane-bound sialidase NEU3 in clathrin-mediated endocytosis.’ → ‘2 text blocks: 1. text block: ‘Role of plasma-membrane-bound sialidase NEU3 in clathrin-mediated endocytosis’, 2. text block: ‘.’)

↓

Remove spaces from end or beginning of text blocks

↓

Split text blocks based on space
‘Role of plasma-membrane-bound sialidase NEU3 in clathrin-mediated endocytosis’
→ ‘Role’ + ‘of’ + ‘plasma-membrane-bound’ + ‘sialidase’
+ ‘NEU3’ + ‘in’ + ‘clathrin-mediated’ + ‘endocytosis’

↓

Search for any closing brackets within a word that are followed by dash and a number
(indicates that the term within the brackets is an abbreviation for the
Split based on brackets
(‘Interleukin(Il)-8’ → ‘Interleukin’ + ‘(‘ + ‘Il’ + ‘)’ + ‘-8’)

↓

Remove apostrophe s from end of word and add as new word
(‘RBD2’s’ + ‘role’ → ‘RBD2’ + ‘’s’ + ‘role’))

Supplementary Fig. S13: Tokenization algorithm.

Supplementary Figure S14

Textmining of PubMed titles and abstracts

SCP specific PubMed article set

↓

Title + abstract text of one article

↓

Tokenize

↓

Stop screening, if text contains any article exclusion words indicating that the investigated biological system is a plant, yeast or bacteria (suppl. table 29)

↓

Search for any key terms that are part of final dictionary
- If one word or a set of words within a sentence, could be part of 2 different key terms, select the longer key term and ignore the other.
- Consider only key terms that are not followed by a key term exclusion word (suppl. table 29).

↓

Define all biological entities that a key term might describe as potential biological entities for that key term.

↓

If a key term is followed by a key term in brackets, consider the key term in brackets as an abbreviation or explanation of the key term in front of the brackets .
→ Set biological entity as the intersection between the potential entities of both key terms.
→ If an intersection does not exist, consider the potential entities of both key terms as potential candidates for the combined key term set.

↓

For any ambiguous key term, i.e. a key term that is related to more than one potential biological entity, search, if there is a different key term for one of the potential biological entities in the same abstract. If yes, consider this entity as the true one.

↓

For any key term that is still ambiguous, count how many abstracts of the same SCP-specific abstract set, contain key terms that un-ambiguously refer to one of the potential entities. Calculate the ratio between these abstract counts and distribute the abstract counts for the ambiguous key term over the identified biological entities according to this ratio.

↓

Ignore any key terms that are still ambiguous at this stage.

↓

Count how many abstracts mention at least one key term of an identified biological entity.

↓

Replace all protein complexes by their subunits and add the # of articles that mention the complex as the # of articles that mention the subunit.

↓

Replace gene family by the family members that were mentioned at least once in the SCP specific abstract set and add the # of articles that mention the family as the # of articles that mention the family member

↓

Biological entity - SCP associations: # of articles that mention a biological entity associated with a SCP

Supplementary Fig. S14: Screening of titles and abstracts of each SCP-specific article for key terms of our final dictionary. We counted the number of articles that mention each biological entity at least once.

Supplementary Figure S15

after 1

Actin filament capping (top 35 of 316 genes) – # of articles that mention a gene associated with a SCP

GSN	57
S100B	17
LRRC16A	16
TMOD1	13
TUBA1A	13
EPS8	11
CD2AP	9
HSPB11	9
CDC42	8
VASP	8
VCL	8
ENAH	7
PNO1	7
PYM1	7
TMOD3	7
CAPZA3	6
HSPA8	6
ING1	6
LOC401913	6
S100A1	6
SH3KBP1	6
TSG1	6
VIM	6
CFL1	5
DNASE1L1	5
DNASE1L3	5
RAC2	5
TTN	5
CAPZA2	4
CAPZB	4
FMN1	4
FMN2	4
GFAP	4
MTPN	4
PROC	4

after 2

Actin filament capping (top 35 of 41 genes) – selectivity in comparison to same level SCPs

GSN	240.7155
LRRC16A	99.06626
S100B	81.11718
TMOD1	64.48416
EPS8	53.89669
CD2AP	51.42693
TMOD3	37.94608
CAPZA3	37.90765
SH3KBP1	35.0775
ENAH	28.80335
S100A1	27.74885
CAPZA2	24.35946
CAPZB	24.35946
MTPN	22.62062
RAC2	19.58641
CAPZA1	18.94322
CFL1	16.6847
SCIN	15.53562
GDI1	14.24983
GDI2	14.06349
ATXN1	13.52871
PGLS	13.52871
S100A2	13.52871
TMOD2	13.52871
DSTN	13.00969
ANXA4	12.75127
TMOD4	12.04326
CD2	11.86524
FMN2	11.3921
FMN1	11.35958
EPB41	10.42482
EPX	9.896251
MSN	8.901432
VASP	8.901432
HSPA9	8.552595

after 3

Actin filament capping – selectivity in comparison to same children set SCPs

S100B	41.13216
LRRC16A	40.32203
TMOD1	26.72763
CD2AP	22.42415
TMOD3	17.9656
CAPZA3	17.6131
S100A1	17.6131
SH3KBP1	17.6131
CAPZA2	11.24676
MTPN	11.24676
CAPZB	10.10528
CAPZA1	8.426579
TMOD4	8.426579
EPS8	7.893277
GDI1	6.030408
GDI2	6.030408
ANXA4	5.612075
ATXN1	5.612075
EPB41	5.612075
EPX	5.612075
HSPA9	5.612075
PGLS	5.612075
S100A2	5.612075
TMOD2	5.612075
CD2	5.515398
ENAH	5.14417
LMOD2	2.803224
ANXA2	1.800782
MSN	1.300643
S100B	41.13216

after 4/5

Actin filament capping – final ranks

LRRC16A	1
TMOD1	2
CD2AP	3
TMOD3	4
CAPZA3	5
SH3KBP1	6
S100A1	7
EPS8	8
CAPZA2	9
MTPN	10.5
CAPZB	10.5
CAPZA1	12
TMOD4	13
TMOD2	14
MSN	15

True positive
False positive
False positive that belongs to sibling SCP

Supplementary Fig. S15: Gene composition of the example SCP Actin filament capping during the computational population pipeline. Shown are the top 35 genes that were associated with the SCP after the indicated population step (see Fig. 2). Genes were ranked by the number of articles within the SCP specific article set that mention that gene (1), by the minus $\log_{10}$(p-values) that were calculated for each gene with all SCPs of the same level as the background set (2), by the minus $\log_{10}$(p-values) that were calculated for each gene with all SCPs of the same children set as the background set (3) and by the final ranks (4). Encircled numbers correspond to the steps of the computational pipeline (Fig. 2). Manually validated true positive genes are labeled blue, false positive genes red and false positive genes that belong to a sibling SCP green.

Supplementary Figure S16

(Normalized) abstract counts (columns) / **(Normalized) abstract counts** (rows)

	**that belong to the gene**	**that belong to all other genes**	
**that belong to the process**	a	b	a + b
**that belong to all other processes**	c	d	c + d
	a + c	b + d	a + b + c + d<br>(Normalized) abstract counts of background set

Supplementary Fig. S16: Contingency table for the calculation of p-values for each gene-SCP association. The background set consists either of all genes that are part of all same level SCPs (same level p-values) or of all genes that are part of all same children set SCPs (same children-set p-value). Notify that the same abstract can be counted multiple times: one time for each identified gene within each SCP.

Supplementary Figure S17

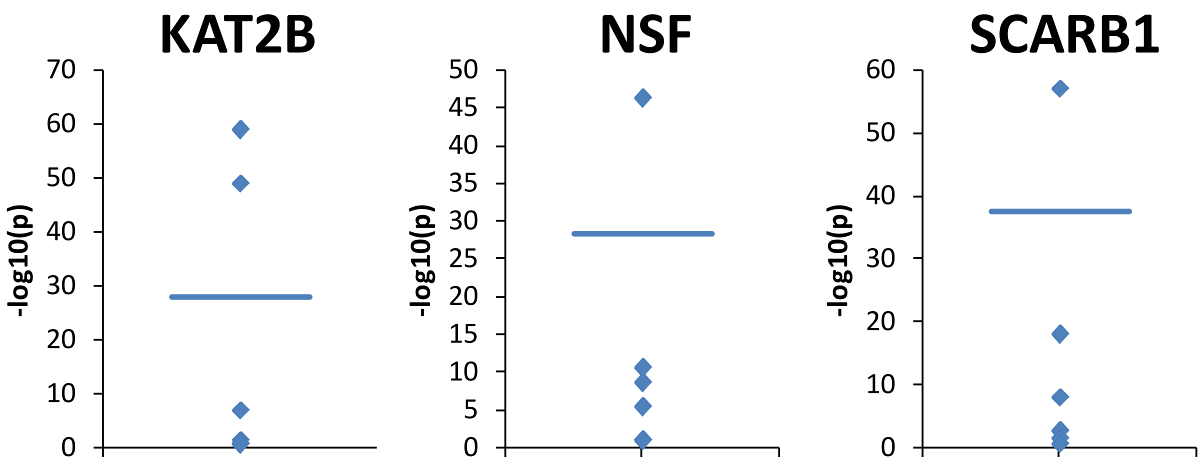

Supplementary Fig. S17: Removal of unselective gene-SCP associations. The minus $\log_{10}$(p-values) of all gene-SCP associations of the same background set (i.e. SCPs of the same level or same children set) were arranged in descending order and the largest gap between any two adjacent minus $\log_{10}$(p-values) was defined to be the selectivity cutoff. Any gene-SCP associations below this cutoff were removed. The Fig. shows the cutoffs (horizontal lines) that were identified for 3 example genes based on all level 3 SCPs as a background. Dots represent the minus log10(p-values) for individual gene-SCP associations.

Supplementary Figure S18

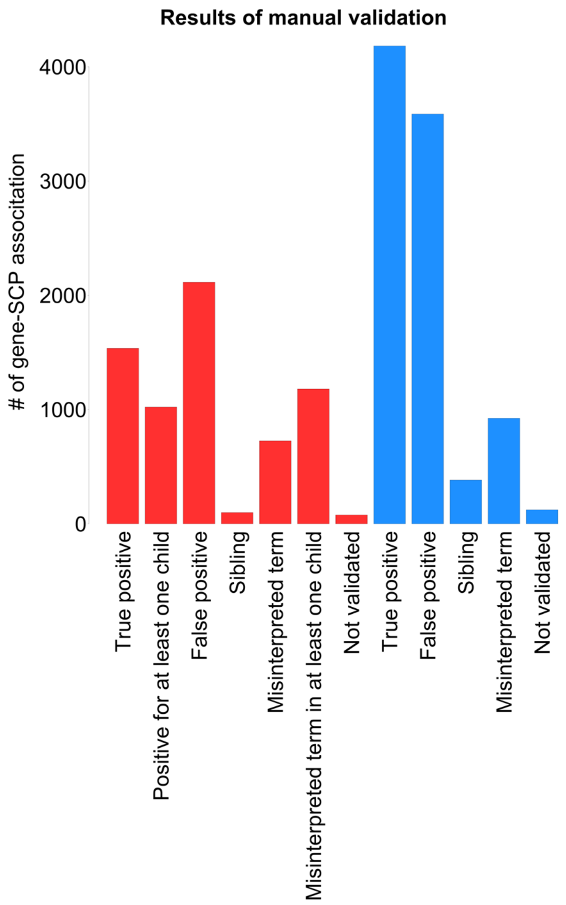

Supplementary Fig. S18: Results of the manual validation. Level-2 (red) and level-3 (blue) gene-SCP associations that were generated by our population algorithm were manually validated, followed by the re-population of the ontology after incorporation of the manual validation results. Since the incorporation of the manual results changed the population results and generated new gene-SCP associations, we repeated the manual validation and following repopulation multiple times until all gene-SCP associations were manually validated. The bar diagram refers to the first populated ontology, i.e. of that ontology that was populated without any manual interference. Not validated gene SCP associations refer to this initial ontology and are removed by the influence of the manually validation results on the population algorithm in the final ontology. Misinterpreted terms label gene-SCP associations that are the result of the misinterpretation of a non-gene term as a gene term by our text mining algorithm. In most cases misinterpreted terms resulted from an incomplete dictionary, so that the addition of these terms to our dictionary will significantly reduce this set of false positives. To reduce manual effort we first validated level-3 gene-SCP associations. For every level-2 gene-SCP association we analyzed, if the gene had been validated as a true positive or a misinterpreted term for any level-3 children SCPs of that particular level-2 SCP. In such cases the manual validation result of the level-3 gene-SCP association was automatically transferred to the level-2 gene-SCP association. During the population of our ontology we favored a low stringency that will generate more true positives with the cost of more false positives, since false positives will be removed during the manual validation.

Supplementary Fig. S19

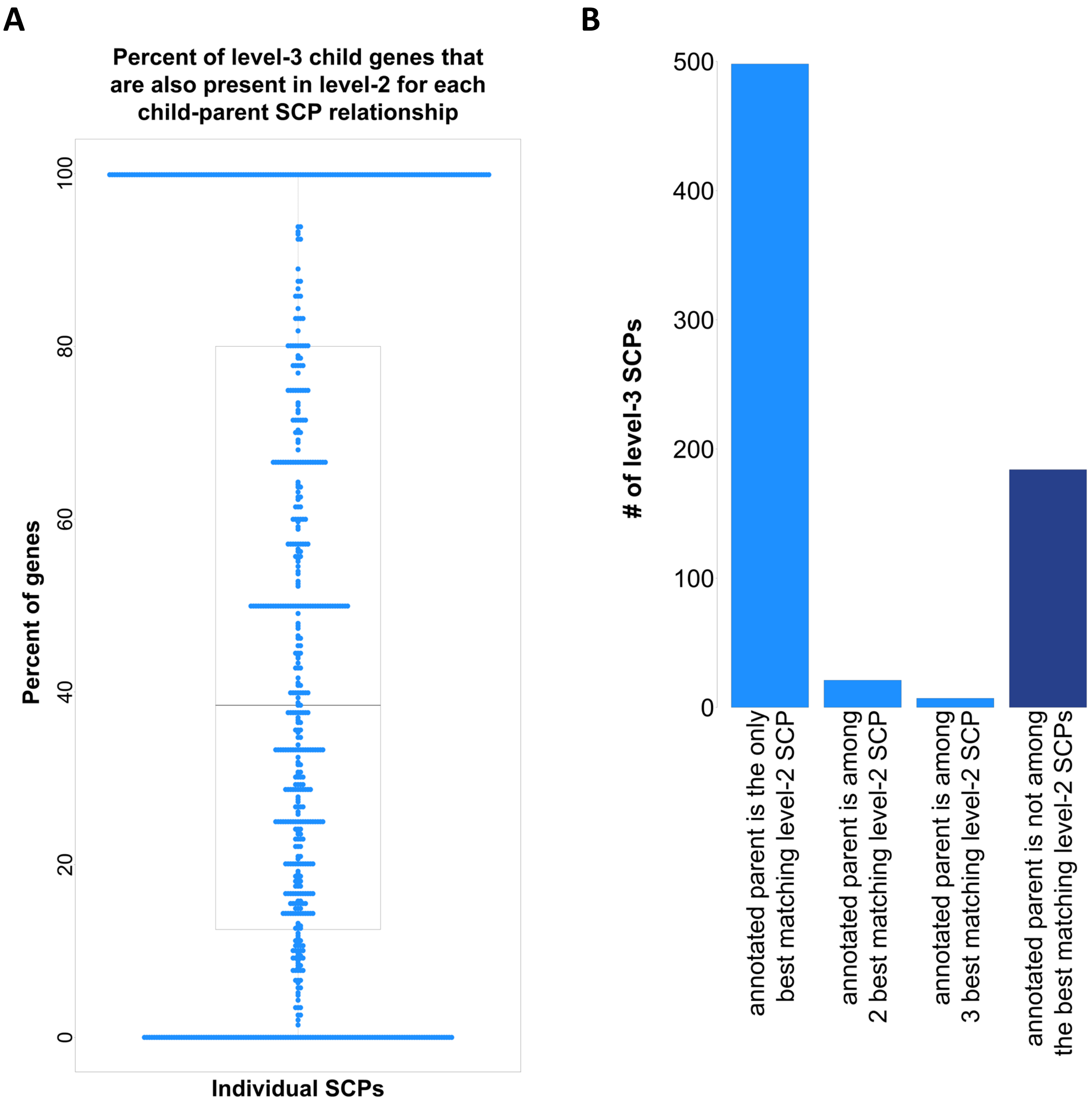

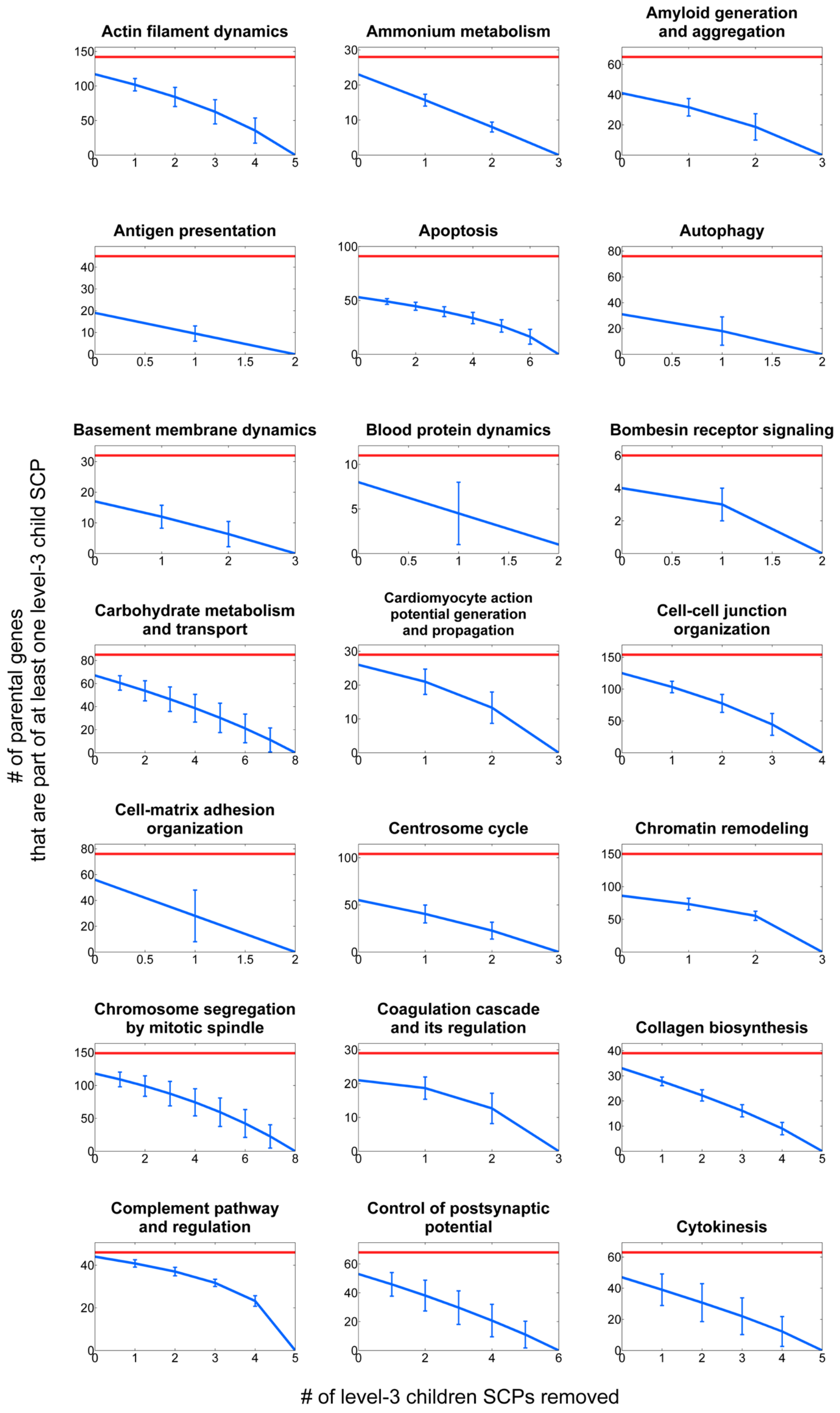

Actin filament dynamics
Ammonium metabolism
Amyloid generation and aggregation
Antigen presentation
Apoptosis
Autophagy
Basement membrane dynamics
Blood protein dynamics
Bombesin receptor signaling
Carbohydrate metabolism and transport
Cardiomyocyte action potential generation and propagation
Cell-cell junction organization
Cell-matrix adhesion organization
Centrosome cycle
Chromatin remodeling
Chromosome segregation by mitotic spindle
Coagulation cascade and its regulation
Collagen biosynthesis
Complement pathway and regulation
Control of postsynaptic potential
Cytokinesis
# of parental genes that are part of at least one level-3 child SCP
# of level-3 children SCPs removed

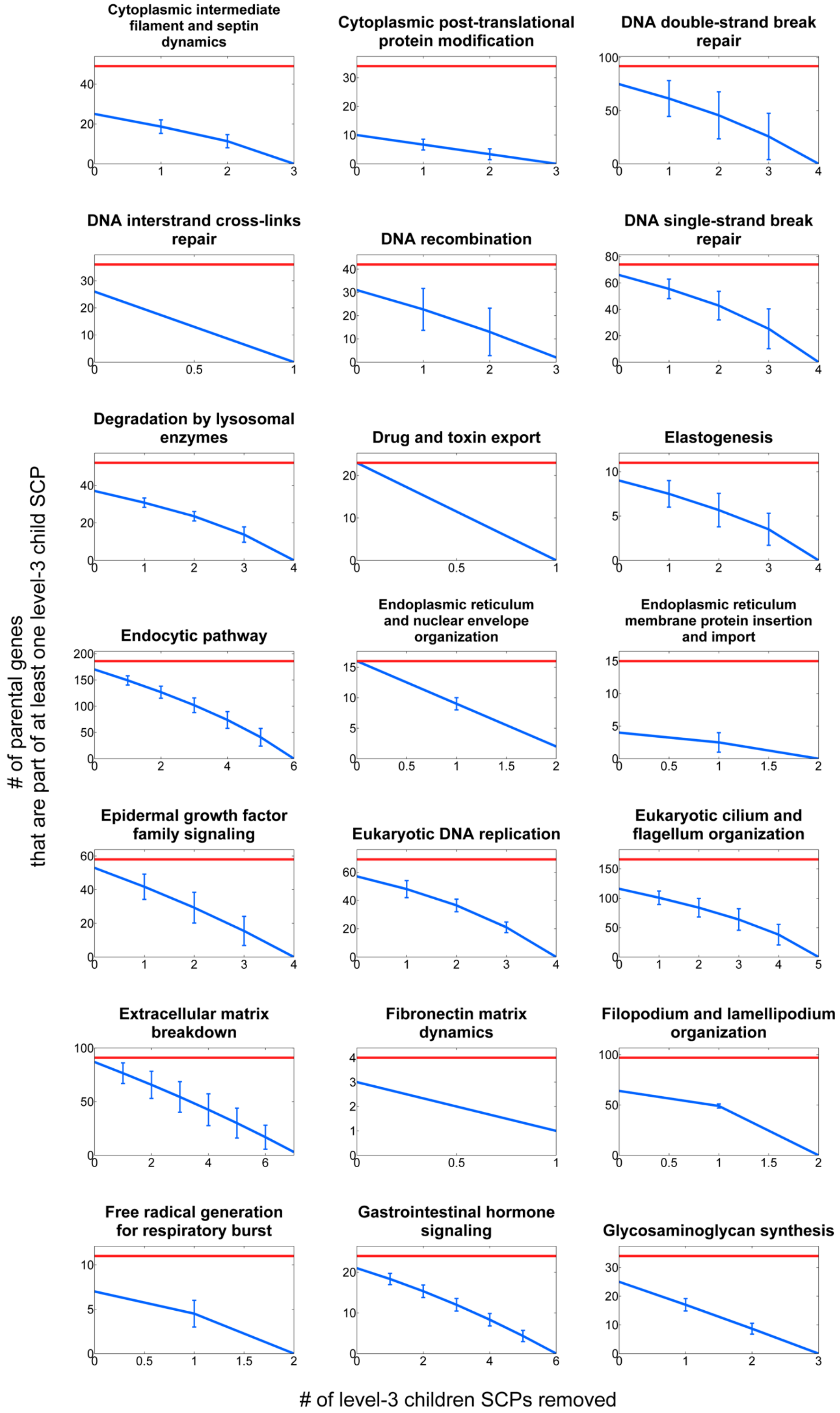
Cytoplasmic intermediate filament and septin dynamics
Cytoplasmic post-translational protein modification
DNA double-strand break repair
DNA interstrand cross-links repair
DNA recombination
DNA single-strand break repair
Degradation by lysosomal enzymes
Drug and toxin export
Elastogenesis
Endocytic pathway
Endoplasmic reticulum and nuclear envelope organization
Endoplasmic reticulum membrane protein insertion and import
Epidermal growth factor family signaling
Eukaryotic DNA replication
Eukaryotic cilium and flagellum organization
Extracellular matrix breakdown
Fibronectin matrix dynamics
Filopodium and lamellipodium organization
Free radical generation for respiratory burst
Gastrointestinal hormone signaling
Glycosaminoglycan synthesis
# of parental genes that are part of at least one level-3 child SCP
# of level-3 children SCPs removed

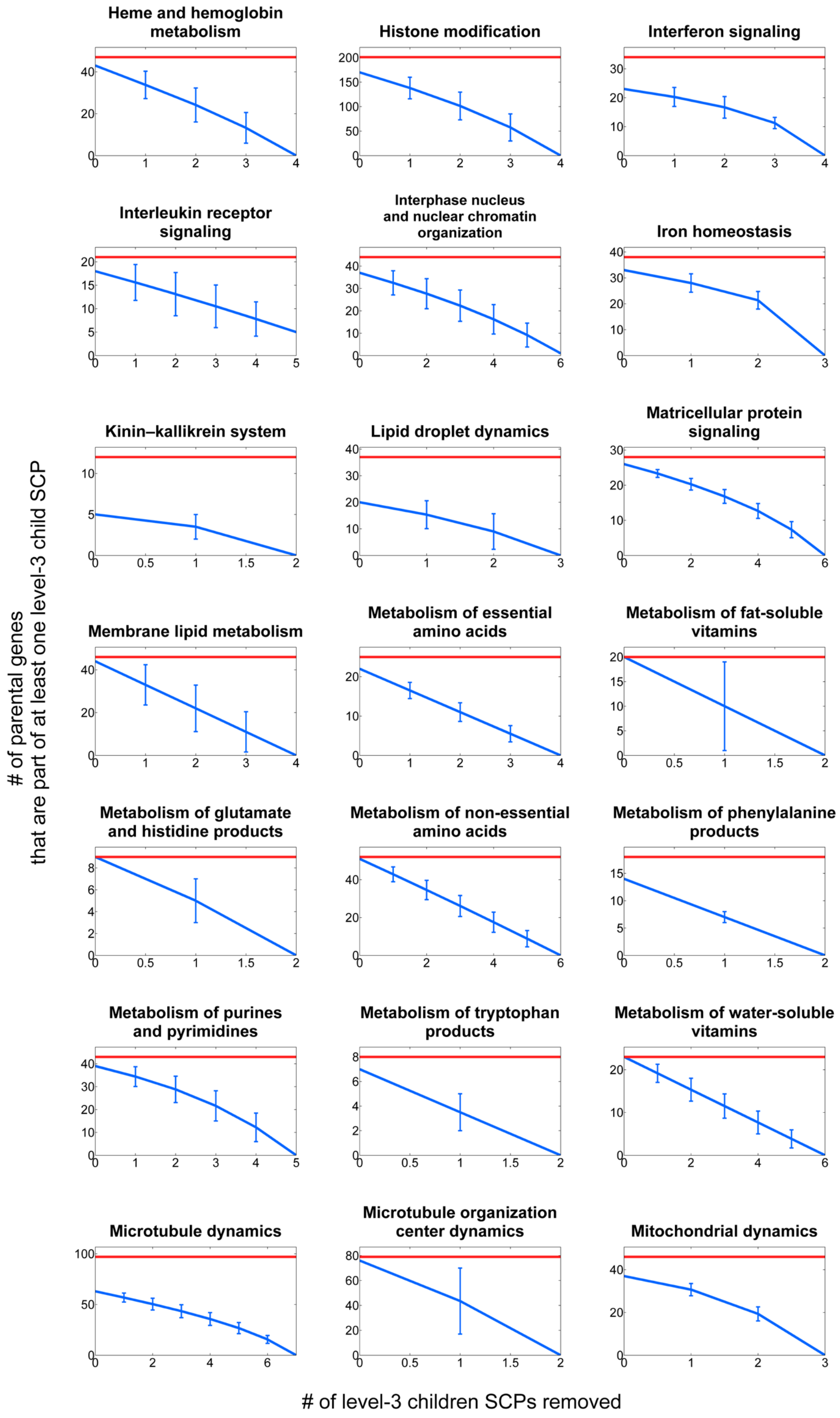
Heme and hemoglobin metabolism
Histone modification
Interferon signaling
Interleukin receptor signaling
Interphase nucleus and nuclear chromatin organization
Iron homeostasis
Kinin–kallikrein system
Lipid droplet dynamics
Matricellular protein signaling
Membrane lipid metabolism
Metabolism of essential amino acids
Metabolism of fat-soluble vitamins
Metabolism of glutamate and histidine products
Metabolism of non-essential amino acids
Metabolism of phenylalanine products
Metabolism of purines and pyrimidines
Metabolism of tryptophan products
Metabolism of water-soluble vitamins
Microtubule dynamics
Microtubule organization center dynamics
Mitochondrial dynamics
# of parental genes that are part of at least one level-3 child SCP
# of level-3 children SCPs removed

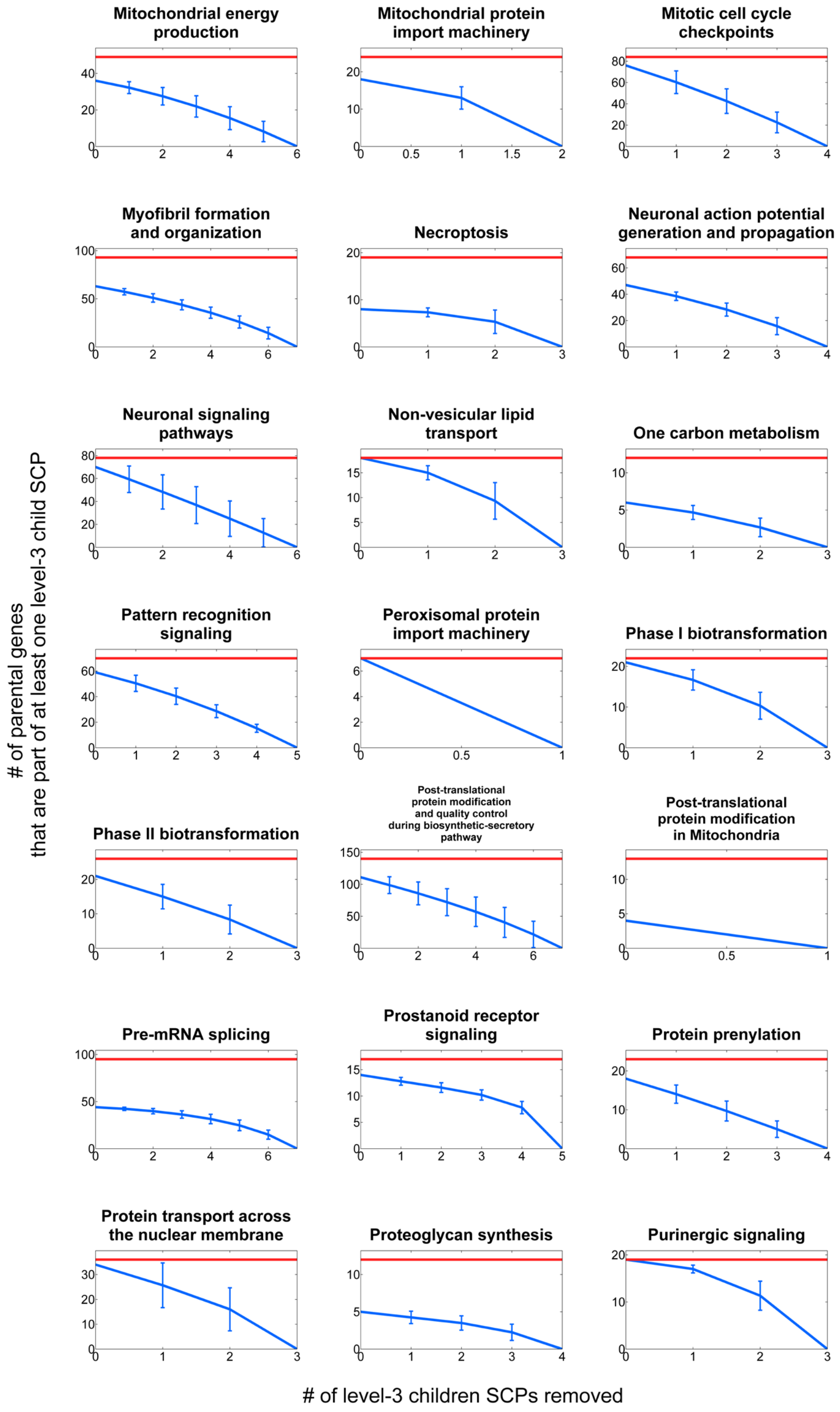
Mitochondrial energy production
Mitochondrial protein import machinery
Mitotic cell cycle checkpoints
Myofibril formation and organization
Necroptosis
Neuronal action potential generation and propagation
Neuronal signaling pathways
Non-vesicular lipid transport
One carbon metabolism
Pattern recognition signaling
Peroxisomal protein import machinery
Phase I biotransformation
Phase II biotransformation
Post-translational protein modification and quality control during biosynthetic-secretory pathway
Post-translational protein modification in Mitochondria
Pre-mRNA splicing
Prostanoid receptor signaling
Protein prenylation
Protein transport across the nuclear membrane
Proteoglycan synthesis
Purinergic signaling
# of parental genes that are part of at least one level-3 child SCP
# of level-3 children SCPs removed

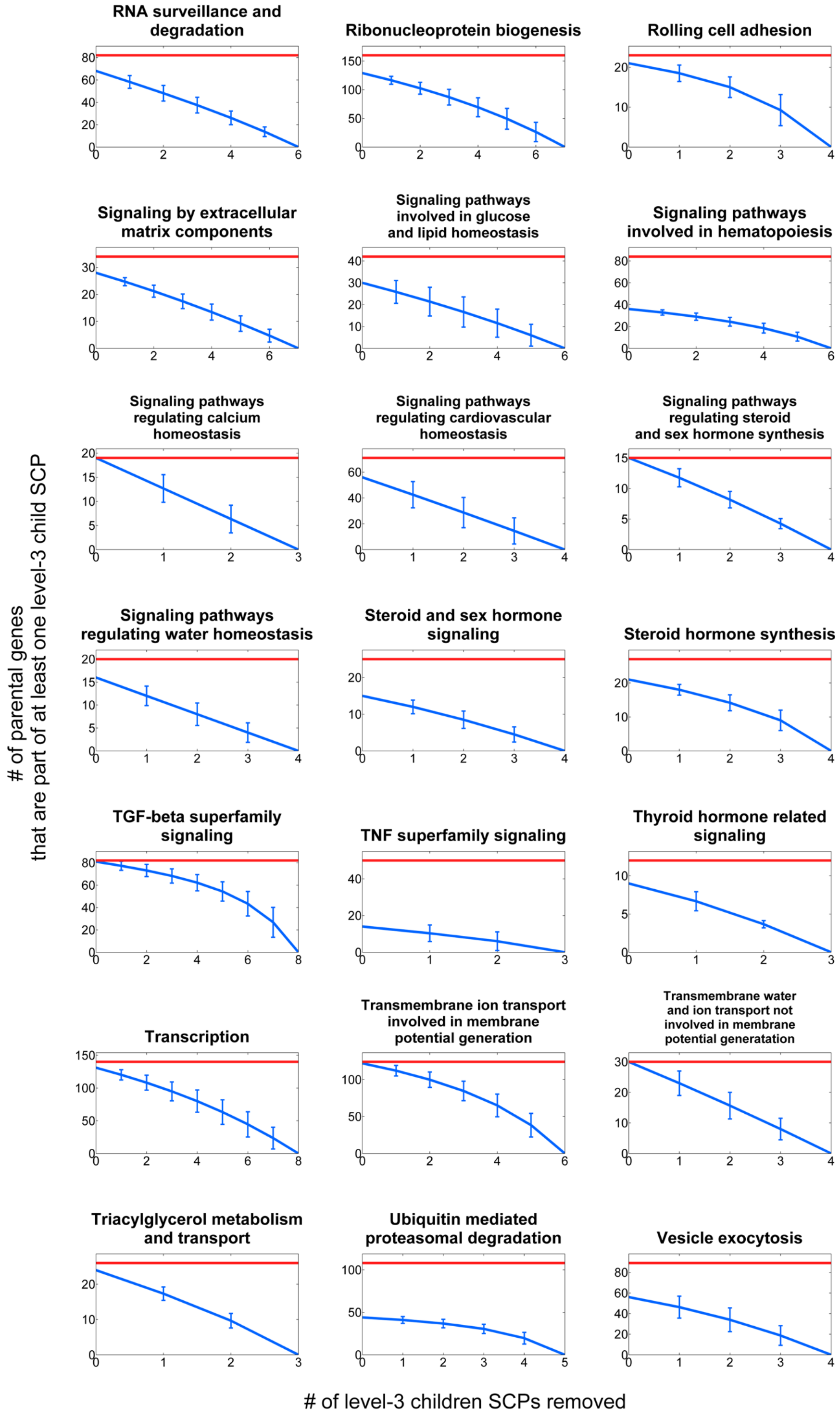
RNA surveillance and degradation
Ribonucleoprotein biogenesis
Rolling cell adhesion
Signaling by extracellular matrix components
Signaling pathways involved in glucose and lipid homeostasis
Signaling pathways involved in hematopoiesis
Signaling pathways regulating calcium homeostasis
Signaling pathways regulating cardiovascular homeostasis
Signaling pathways regulating steroid and sex hormone synthesis
Signaling pathways regulating water homeostasis
Steroid and sex hormone signaling
Steroid hormone synthesis
TGF-beta superfamily signaling
TNF superfamily signaling
Thyroid hormone related signaling
Transcription
Transmembrane ion transport involved in membrane potential generation
Transmembrane water and ion transport not involved in membrane potential generatation
Triacylglycerol metabolism and transport
Ubiquitin mediated proteasomal degradation
Vesicle exocytosis
# of parental genes that are part of at least one level-3 child SCP
# of level-3 children SCPs removed

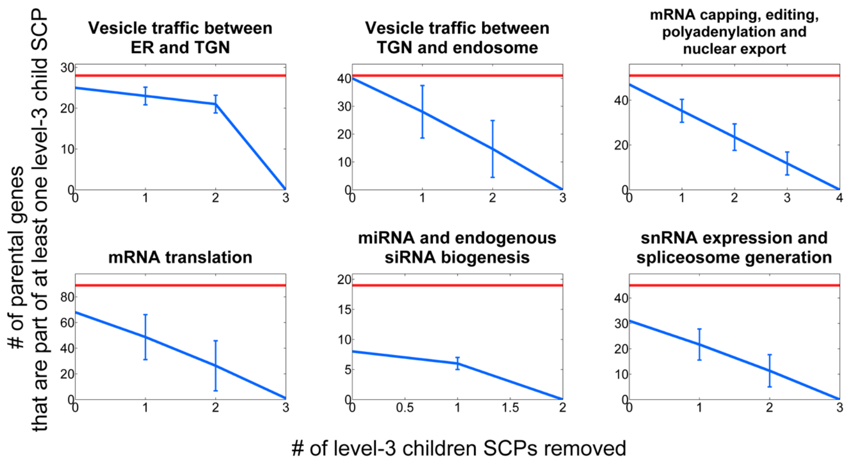

Supplementary Fig. S19: Analysis of the populated ontology. (A) For each level-3 SCP we calculated the percentage of genes that were also part of its annotated level-2 parent SCP. Results were visualized as a box plot, each dot describes the percentage of overlapping genes for one level-3 SCP. This analysis was done before addition of the genes of level-3 SCPs to their level-2 parent SCPs. (B) For each level-3 child SCP we screened all level-2 SCPs for that SCP that contains most of the level-3 SCP's genes. For 503 level-3 SCPs the identified level-2 SCP was the annotated parent SCP, for 21 and 7 level-3 SCPs we identified two and three best matching level-2 SCPs that contained the annotated parent SCP. For 181 level-3 SCP the annotated parent SCP was not among the best matching SCPs. (C) The number of overlapping genes between each level-2 parent SCP and the union of all its level-3 children SCPs was determined. For each parent at a time, the indicated number of its children SCPs was removed, followed by the re-population of the remaining level-3 SCPs and the recalculation of the number of overlapping genes between that parent and its remaining children SCPs. All possible combinations of removed children were considered. Solid blue lines indicate the average overlap, error bars the standard deviation. Solid light red lines indicate the total number of genes of the parent SCP.

Supplementary Figure S20

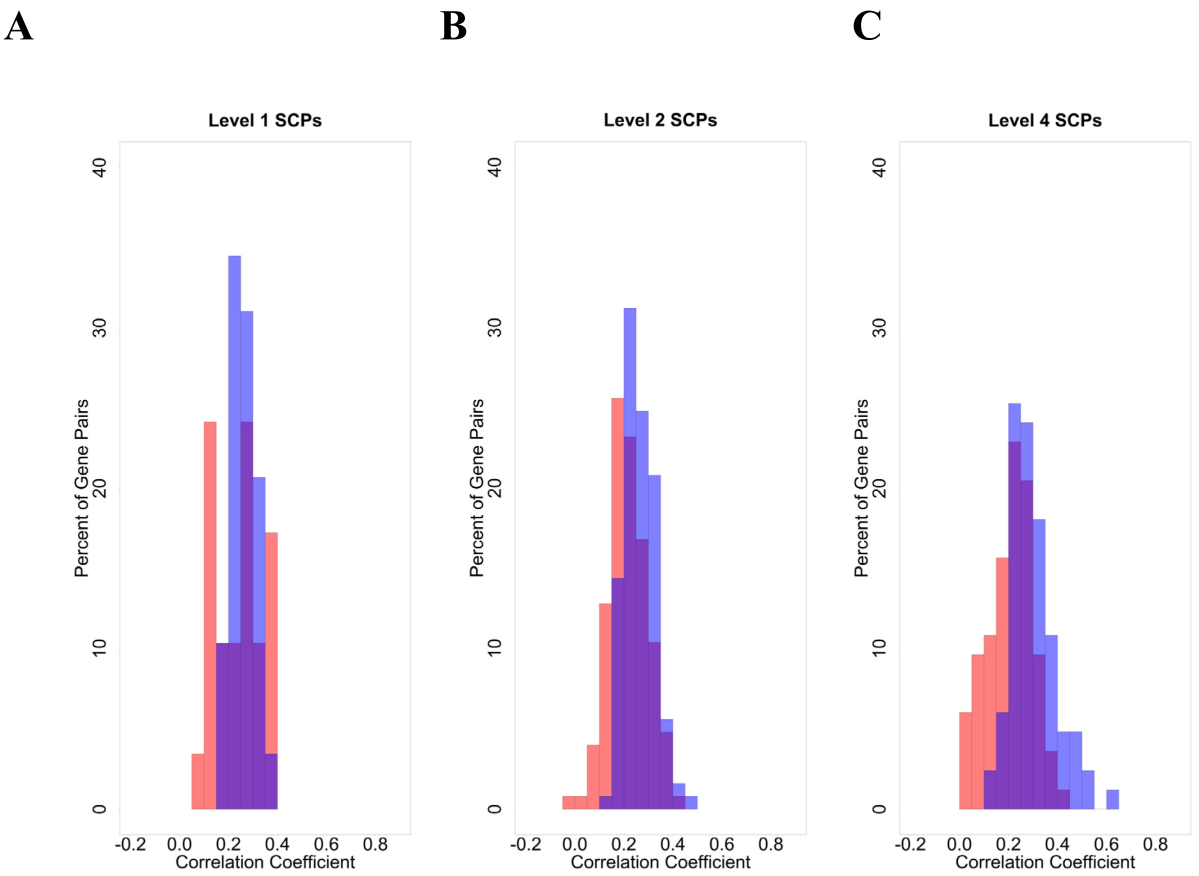

Supplementary Fig. S20: Correlation between gene pairs. The correlation between gene pairs of (A) level-1, (B) level-2 and (C) level-4 SCPs were obtained as described in Fig. 4. Kolmogorov-Smirnov test p-values are 0.156, 1.53e-05, and 2.74e-04 respectively.

Supplementary Figure S21

A

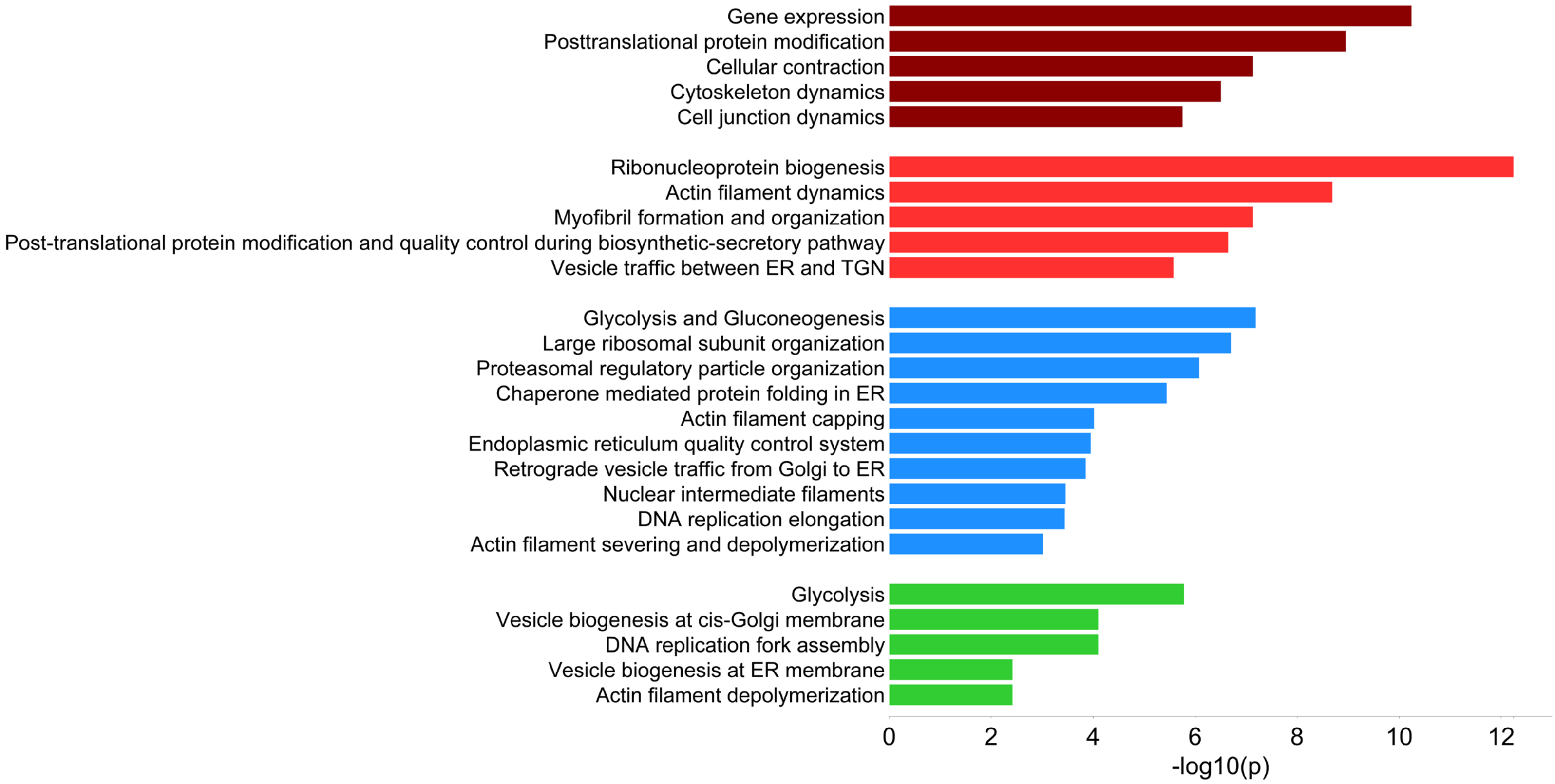

B

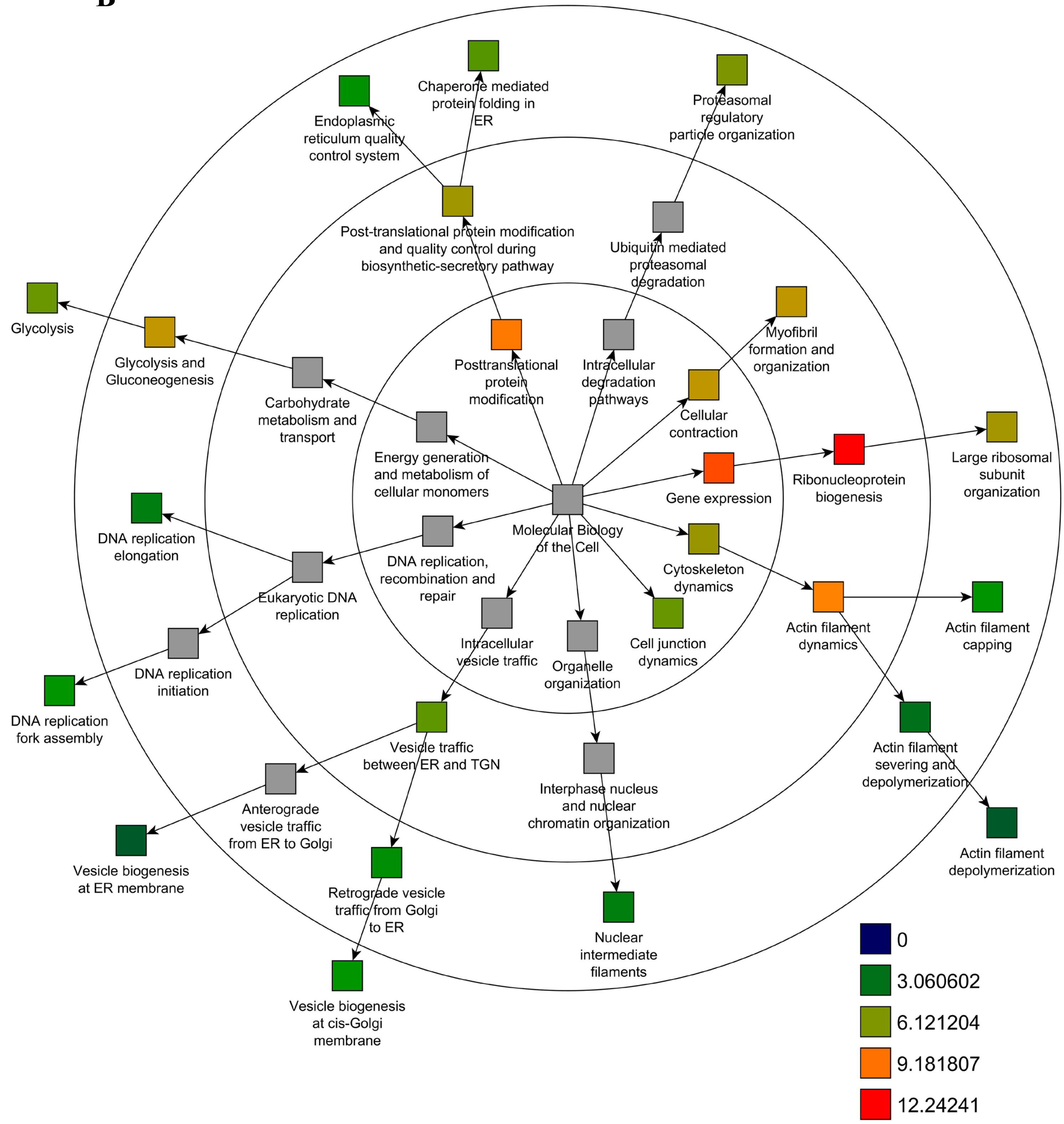
Chaperone mediated protein folding in ER
Endoplasmic reticulum quality control system
Proteasomal regulatory particle organization
Post-translational protein modification and quality control during biosynthetic-secretory pathway
Ubiquitin mediated proteasomal degradation
Glycolysis
Glycolysis and Gluconeogenesis
Myofibril formation and organization
Posttranslational protein modification
Intracellular degradation pathways
Carbohydrate metabolism and transport
Cellular contraction
Large ribosomal subunit organization
Energy generation and metabolism of cellular monomers
Ribonucleoprotein biogenesis
Gene expression
DNA replication elongation
Molecular Biology of the Cell
DNA replication, recombination and repair
Cytoskeleton dynamics
Eukaryotic DNA replication
Intracellular vesicle traffic
Cell junction dynamics
Actin filament dynamics
Actin filament capping
Organelle organization
DNA replication initiation
DNA replication fork assembly
Vesicle traffic between ER and TGN
Actin filament severing and depolymerization
Interphase nucleus and nuclear chromatin organization
Anterograde vesicle traffic from ER to Golgi
Vesicle biogenesis at ER membrane
Actin filament depolymerization
Retrograde vesicle traffic from Golgi to ER
Nuclear intermediate filaments
Vesicle biogenesis at cis-Golgi membrane
0
3.060602
6.121204
9.181807
12.24241

C

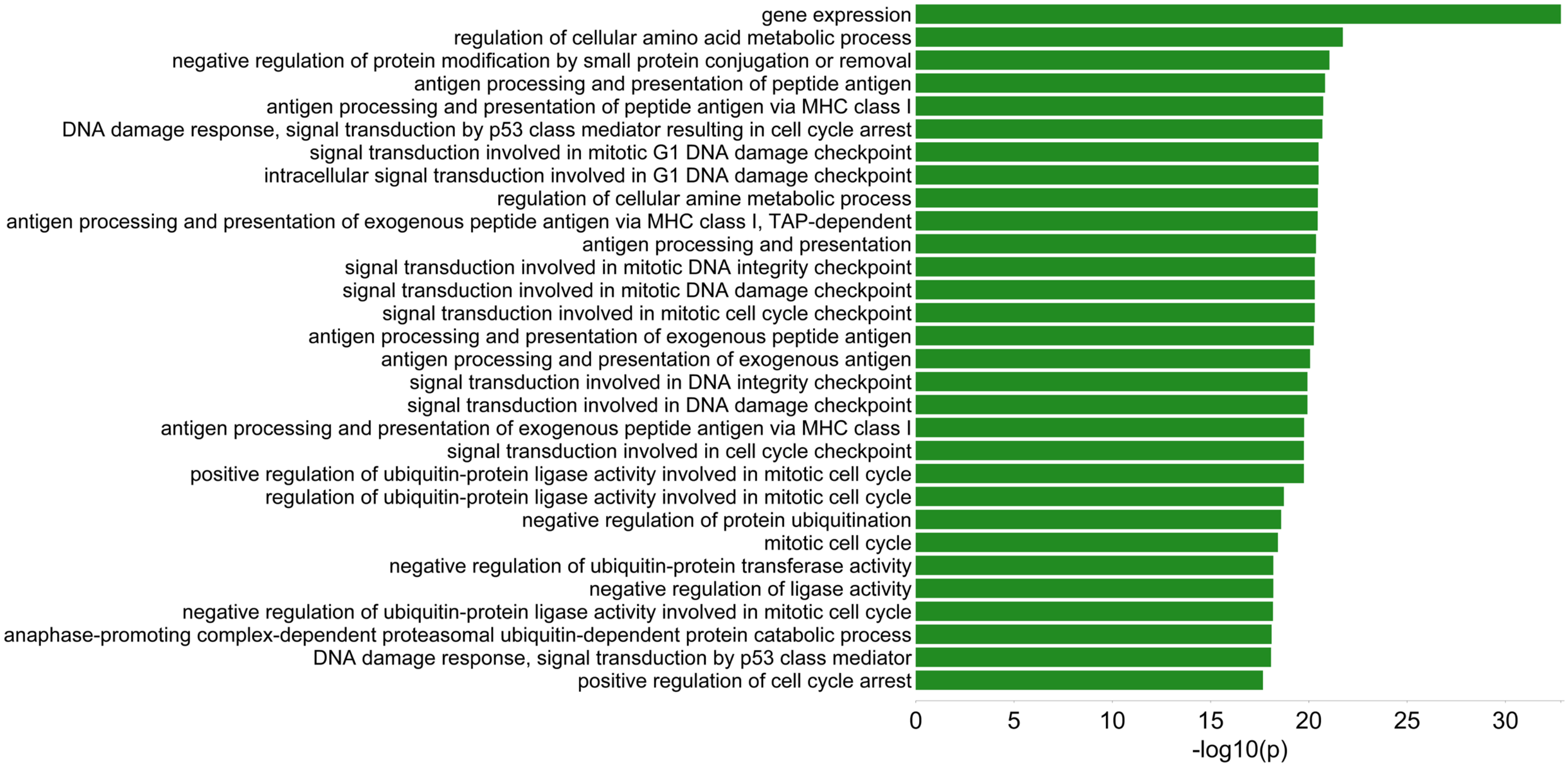
gene expression
regulation of cellular amino acid metabolic process
negative regulation of protein modification by small protein conjugation or removal
antigen processing and presentation of peptide antigen
antigen processing and presentation of peptide antigen via MHC class I
DNA damage response, signal transduction by p53 class mediator resulting in cell cycle arrest
signal transduction involved in mitotic G1 DNA damage checkpoint
intracellular signal transduction involved in G1 DNA damage checkpoint
regulation of cellular amine metabolic process
antigen processing and presentation of exogenous peptide antigen via MHC class I, TAP-dependent
antigen processing and presentation
signal transduction involved in mitotic DNA integrity checkpoint
signal transduction involved in mitotic DNA damage checkpoint
signal transduction involved in mitotic cell cycle checkpoint
antigen processing and presentation of exogenous peptide antigen
antigen processing and presentation of exogenous antigen
signal transduction involved in DNA integrity checkpoint
signal transduction involved in DNA damage checkpoint
antigen processing and presentation of exogenous peptide antigen via MHC class I
signal transduction involved in cell cycle checkpoint
positive regulation of ubiquitin-protein ligase activity involved in mitotic cell cycle
regulation of ubiquitin-protein ligase activity involved in mitotic cell cycle
negative regulation of protein ubiquitination
mitotic cell cycle
negative regulation of ubiquitin-protein transferase activity
negative regulation of ligase activity
negative regulation of ubiquitin-protein ligase activity involved in mitotic cell cycle
anaphase-promoting complex-dependent proteasomal ubiquitin-dependent protein catabolic process
DNA damage response, signal transduction by p53 class mediator
positive regulation of cell cycle arrest
0
5
10
15
20
25
30
-log10(p)

D

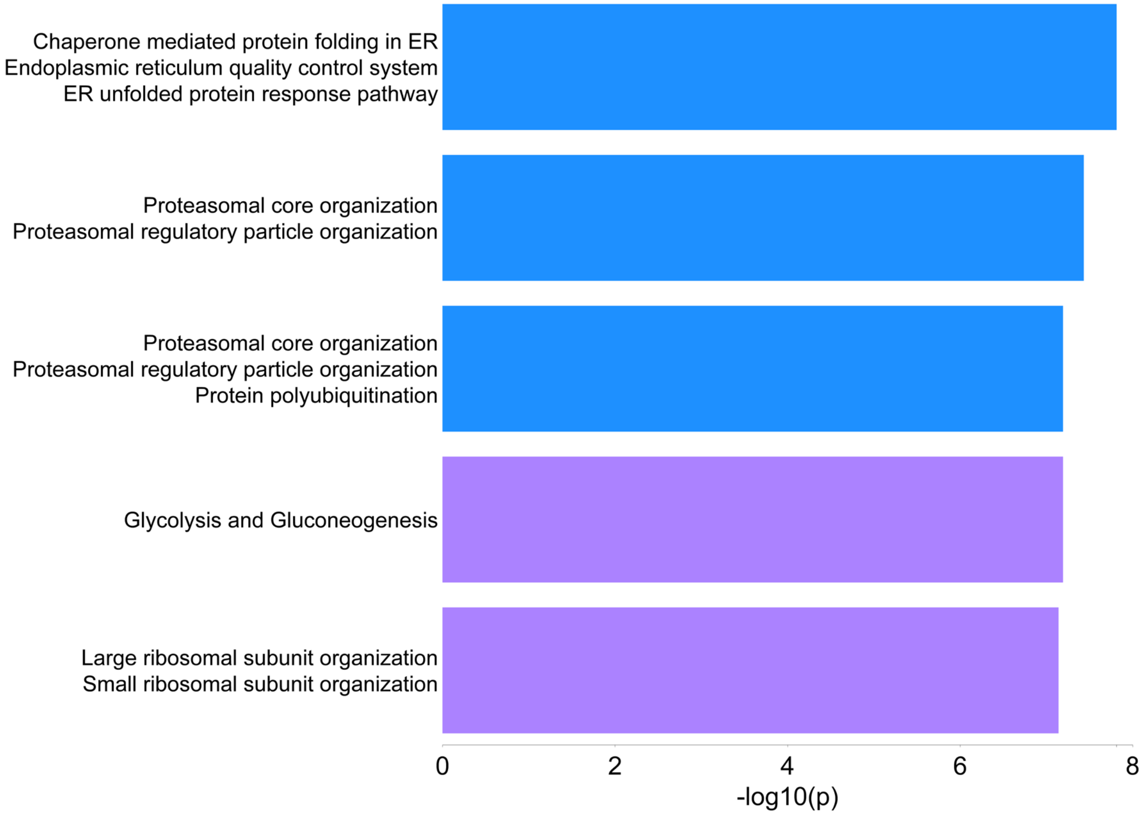
Chaperone mediated protein folding in ER
Endoplasmic reticulum quality control system
ER unfolded protein response pathway
Proteasomal core organization
Proteasomal regulatory particle organization
Proteasomal core organization
Proteasomal regulatory particle organization
Protein polyubiquitination
Glycolysis and Gluconeogenesis
Large ribosomal subunit organization
Small ribosomal subunit organization
0
2
4
6
8
-log10(p)

E

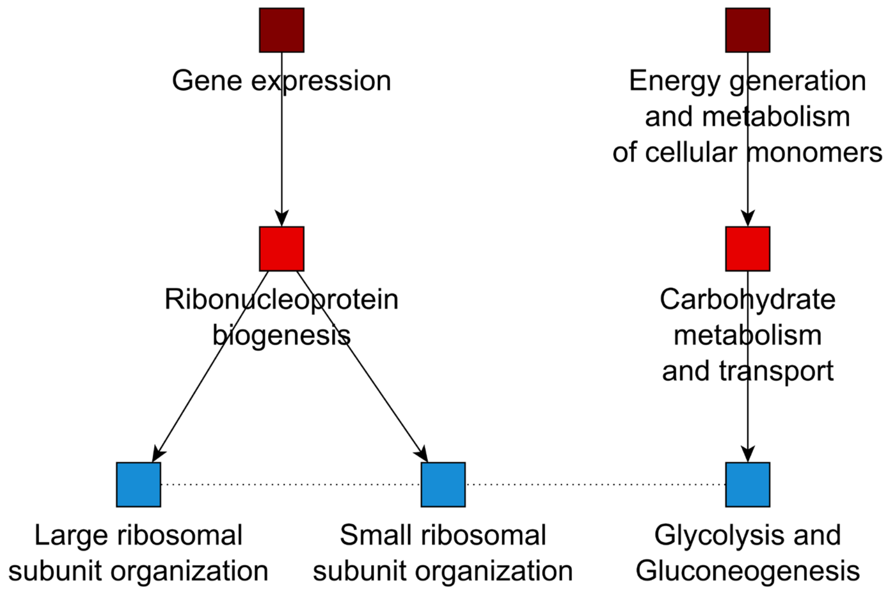

Supplementary Fig. S21: SCPs identified by standard and dynamic enrichment analysis of proteins that were identified as gained or lost interaction partners of mutant CFTR as determined by co-immunoprecipitation followed by proteomic analysis. (A) Proteins were subjected to standard enrichment analysis via Fisher's Exact test. Shown are the top 5 level-1, level-2 and level-4 as well as the top 10 level-3 SCPs that were predicted to be regulated by the identified protein interaction partners. Bars indicate minus $\log_{10}$(p-values). **(B)** Annotated parent-child relationships between the predicted SCP. Colors indicate minus $\log_{10}$(p-values). SCPs in gray were not among the top predicted SCPs but are descendents of SCPs that were among the top predictions. **(C)** Top 30 predicted Gene Ontology biological processes that were identified based on standard enrichment analysis. **(D)** Top 5 predicted SCPs or SCP units that were identified via dynamic enrichment analysis. See Fig. 5 for details. Blue bars: minus $\log_{10}$ (p-values) of those single SCPs or SCP-units that are part of the largest SCP networks shown in Fig. 5. Purple bars: minus $\log_{10}$ (p-values) of all other predictions. **(E)** Predicted level-3 SCPs (light blue) that were not part of the largest SCP network (violet bar in Supplementary Fig. S21D) and their annotated level-1 grandparent and level-2 parent SCPs.

Supplementary Fig. S22

A

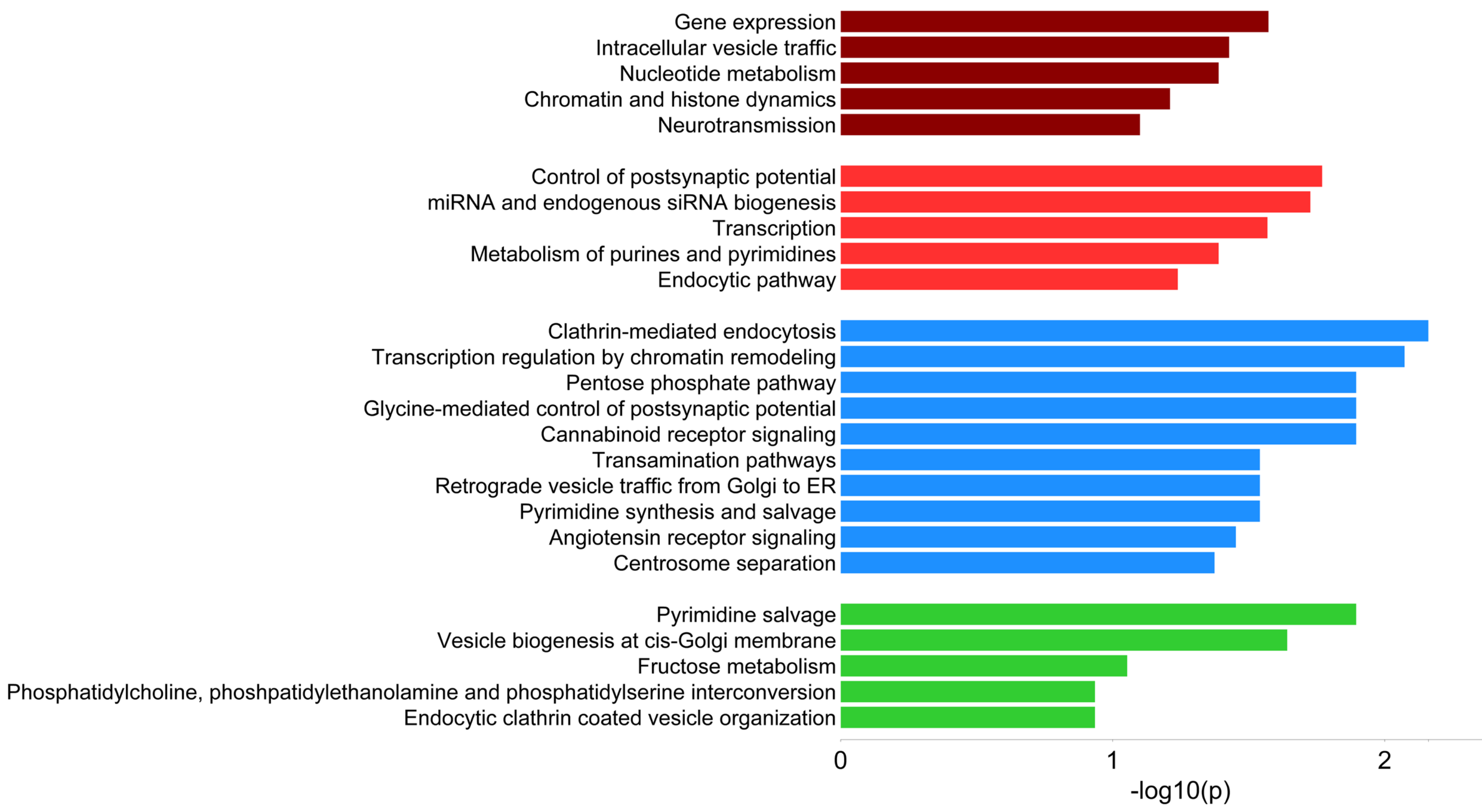

B

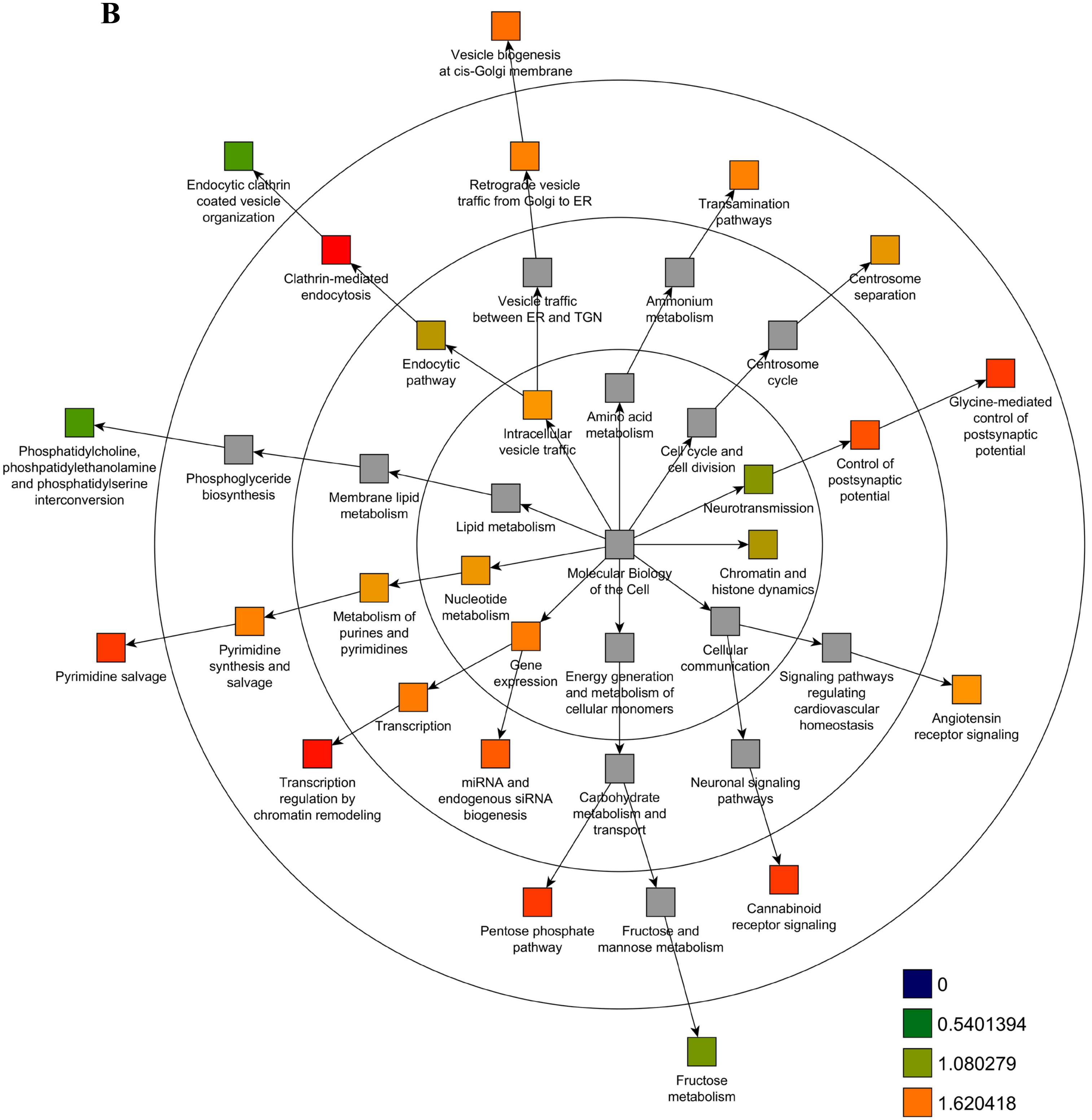
Vesicle biogenesis at cis-Golgi membrane
Endocytic clathrin coated vesicle organization
Retrograde vesicle traffic from Golgi to ER
Transamination pathways
Centrosome separation
Clathrin-mediated endocytosis
Vesicle traffic between ER and TGN
Ammonium metabolism
Endocytic pathway
Centrosome cycle
Glycine-mediated control of postsynaptic potential
Phosphatidylcholine, phoshpatidylethanolamine and phosphatidylserine interconversion
Intracellular vesicle traffic
Amino acid metabolism
Cell cycle and cell division
Control of postsynaptic potential
Phosphoglyceride biosynthesis
Membrane lipid metabolism
Neurotransmission
Lipid metabolism
Molecular Biology of the Cell
Chromatin and histone dynamics
Nucleotide metabolism
Metabolism of purines and pyrimidines
Pyrimidine synthesis and salvage
Pyrimidine salvage
Gene expression
Energy generation and metabolism of cellular monomers
Cellular communication
Signaling pathways regulating cardiovascular homeostasis
Angiotensin receptor signaling
Transcription
Transcription regulation by chromatin remodeling
miRNA and endogenous siRNA biogenesis
Carbohydrate metabolism and transport
Neuronal signaling pathways
Cannabinoid receptor signaling
Pentose phosphate pathway
Fructose and mannose metabolism
Fructose metabolism
0
0.5401394
1.080279
1.620418
2.160558

C

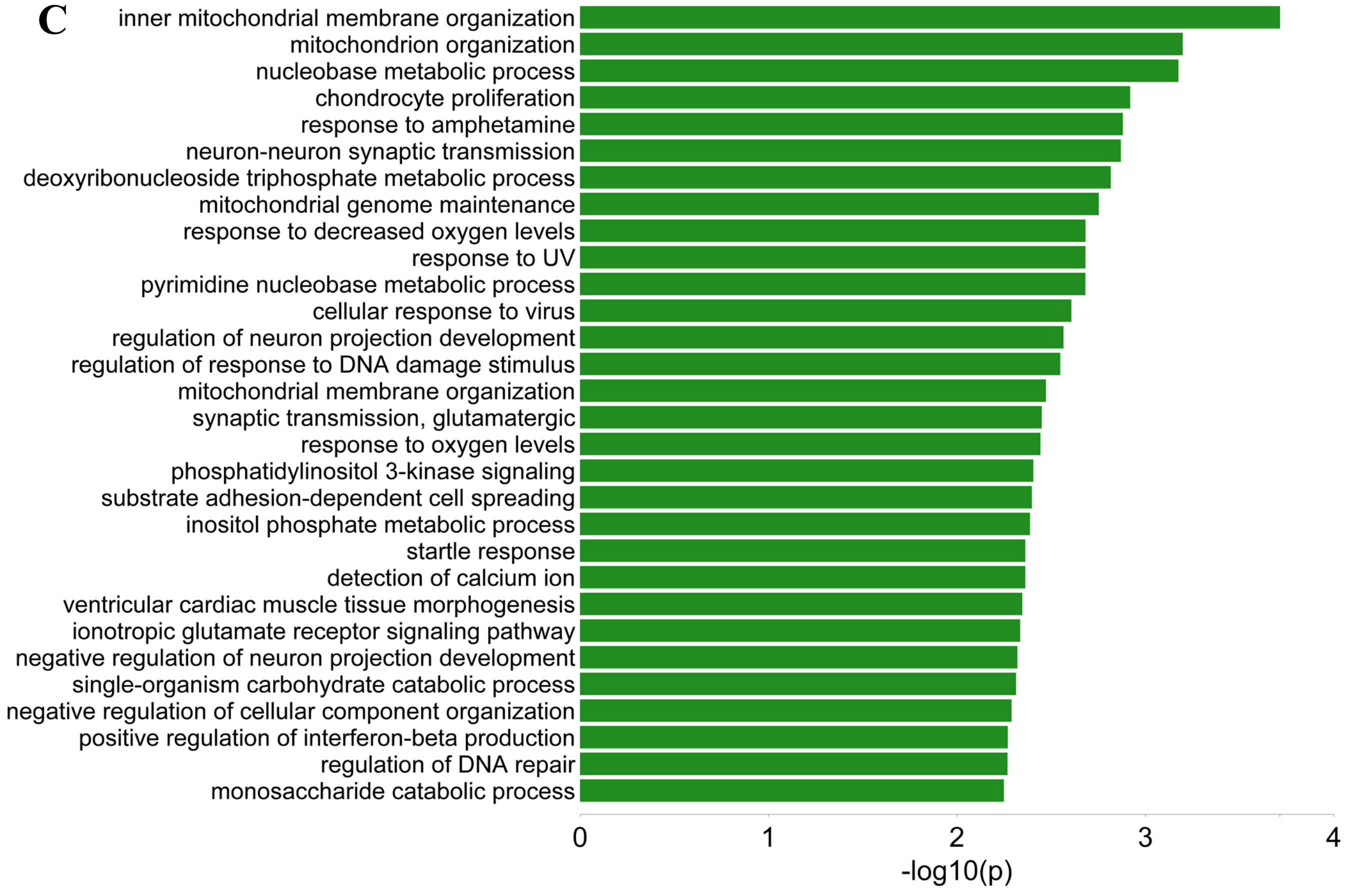

inner mitochondrial membrane organization
mitochondrion organization
nucleobase metabolic process
chondrocyte proliferation
response to amphetamine
neuron-neuron synaptic transmission
deoxyribonucleoside triphosphate metabolic process
mitochondrial genome maintenance
response to decreased oxygen levels
response to UV
pyrimidine nucleobase metabolic process
cellular response to virus
regulation of neuron projection development
regulation of response to DNA damage stimulus
mitochondrial membrane organization
synaptic transmission, glutamatergic
response to oxygen levels
phosphatidylinositol 3-kinase signaling
substrate adhesion-dependent cell spreading
inositol phosphate metabolic process
startle response
detection of calcium ion
ventricular cardiac muscle tissue morphogenesis
ionotropic glutamate receptor signaling pathway
negative regulation of neuron projection development
single-organism carbohydrate catabolic process
negative regulation of cellular component organization
positive regulation of interferon-beta production
regulation of DNA repair
monosaccharide catabolic process
0
1
2
3
4
-log10(p)

D

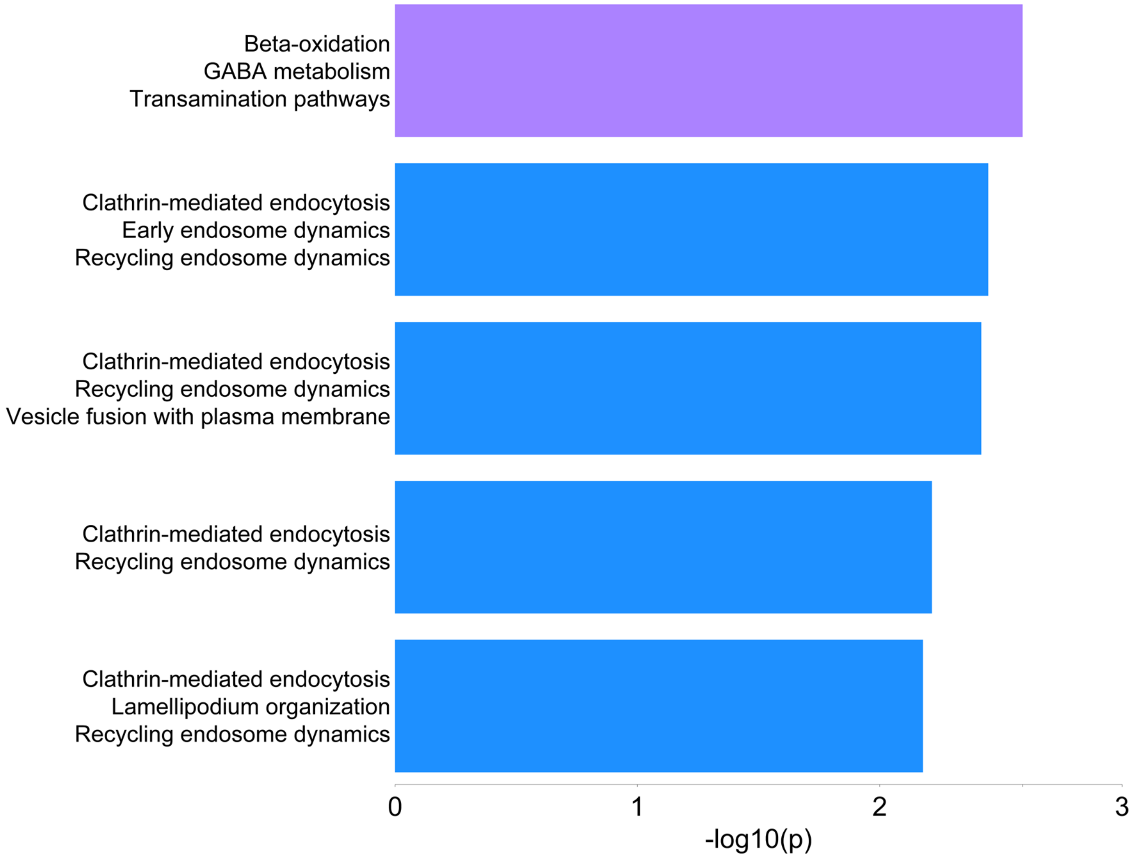

Beta-oxidation
GABA metabolism
Transamination pathways
Clathrin-mediated endocytosis
Early endosome dynamics
Recycling endosome dynamics
Clathrin-mediated endocytosis
Recycling endosome dynamics
Vesicle fusion with plasma membrane
Clathrin-mediated endocytosis
Recycling endosome dynamics
Clathrin-mediated endocytosis
Lamellipodium organization
Recycling endosome dynamics
0
1
2
3
-log10(p)

E

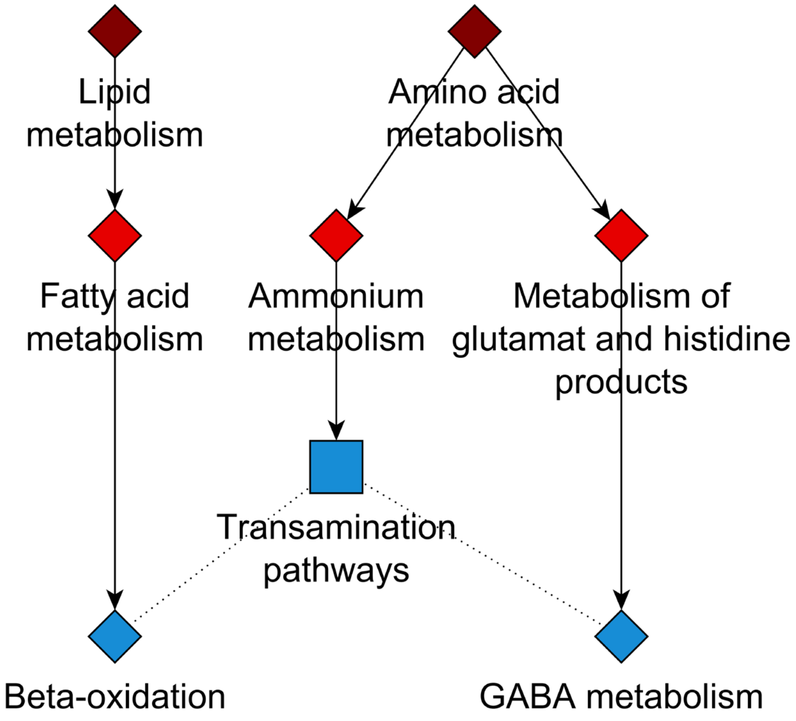

Supplementary Fig. S22: SCPs identified by standard and dynamic enrichment analysis of genes that were identified as regulators of the secretory pathway. (A) Genes were subjected to standard enrichment analysis via Fisher's Exact test. Shown are the top 5 level-1, level-2 and level-4 as well as the top 10 level-3 SCPs that were predicted to be regulated by the identified protein interaction partners. Bars indicate minus log_{10}(p-values). **(B)** Annotated parent-child relationships between the predicted SCP. Colors indicate minus log_{10}(p-values). SCPs in gray were not among the top predicted SCPs but are descendents of SCPs that were among the top predictions. **(C)** Top 30 predicted Gene Ontology biological processes that were identified based on standard enrichment analysis.**(D)** Top 5 predicted SCPs or SCP units that were identified via dynamic enrichment analysis. See Fig. 5 for details. Blue bars: minus log_{10} (p-values) of those single SCPs or SCP-units that are part of the largest SCP networks shown in Fig. 5. Purple bars: minus log_{10} (p-values) of all other predictions. **(E)** Predicted level-3 SCPs (light blue) that were not part of the largest SCP network (violet bar in Supplementary Fig. S21D) and their annotated level-1 grandparent and level-2 parent SCPs.

Supplementary Fig. S23

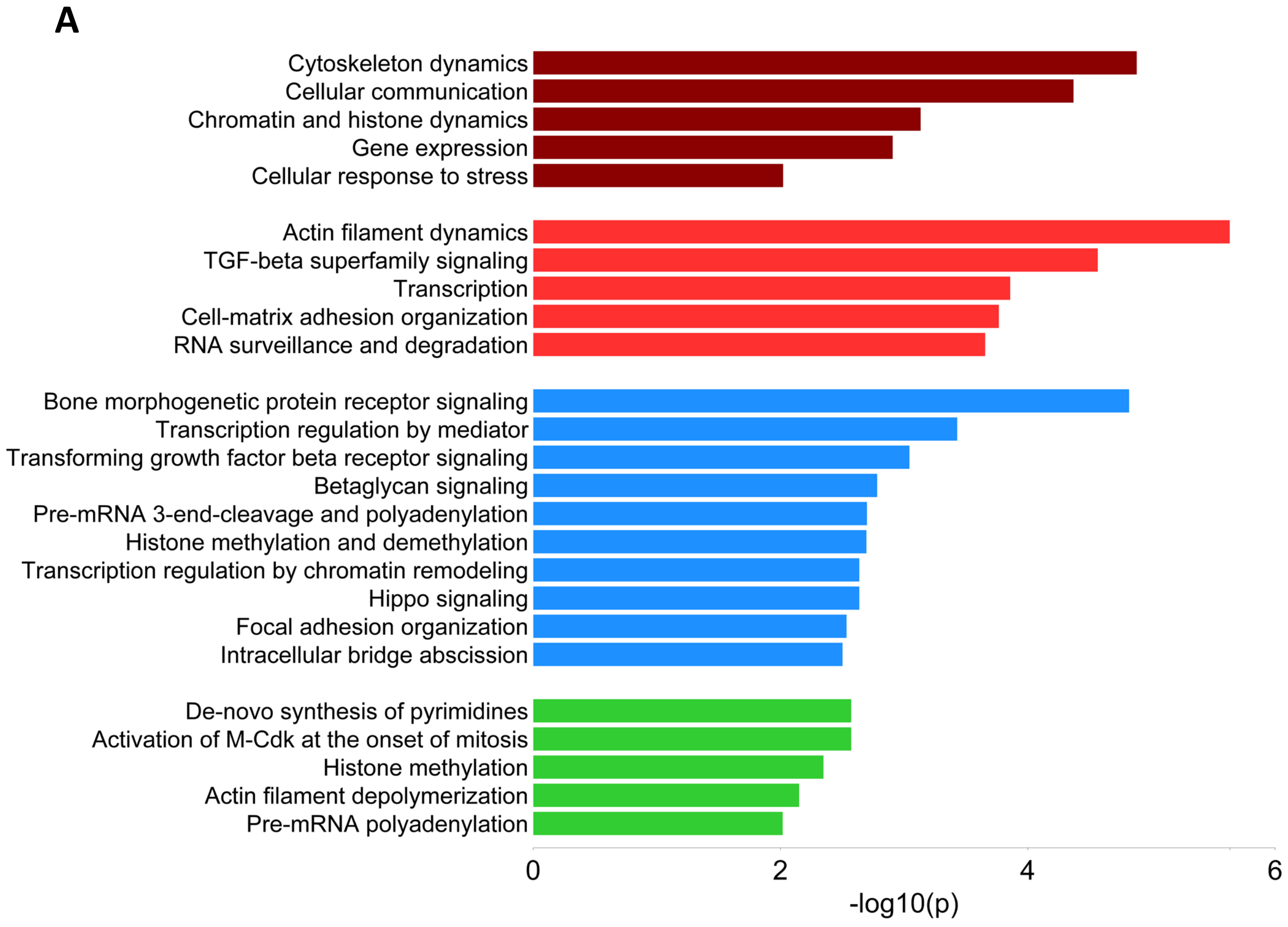

B

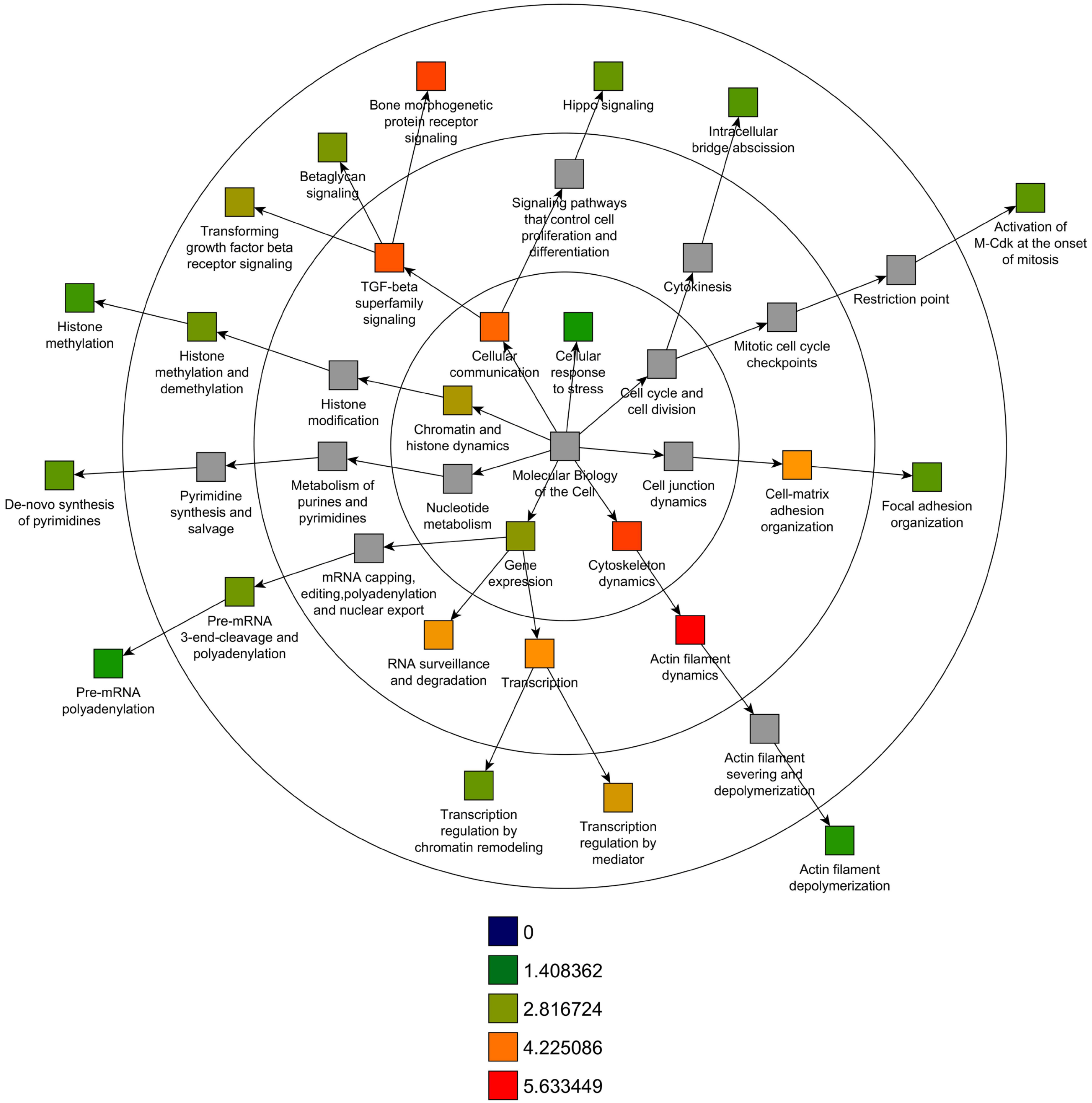
Bone morphogenetic protein receptor signaling
Hippo signaling
Intracellular bridge abscission
Betaglycan signaling
Signaling pathways that control cell proliferation and differentiation
Activation of M-Cdk at the onset of mitosis
Transforming growth factor beta receptor signaling
TGF-beta superfamily signaling
Cytokinesis
Restriction point
Histone methylation
Histone methylation and demethylation
Cellular communication
Cellular response to stress
Mitotic cell cycle checkpoints
Histone modification
Chromatin and histone dynamics
Cell cycle and cell division
De-novo synthesis of pyrimidines
Pyrimidine synthesis and salvage
Metabolism of purines and pyrimidines
Nucleotide metabolism
Molecular Biology of the Cell
Cell junction dynamics
Cell-matrix adhesion organization
Focal adhesion organization
mRNA capping, editing,polyadenylation and nuclear export
Gene expression
Cytoskeleton dynamics
Pre-mRNA 3-end-cleavage and polyadenylation
Pre-mRNA polyadenylation
RNA surveillance and degradation
Transcription
Actin filament dynamics
Actin filament severing and depolymerization
Transcription regulation by chromatin remodeling
Transcription regulation by mediator
Actin filament depolymerization
0
1.408362
2.816724
4.225086
5.633449

C

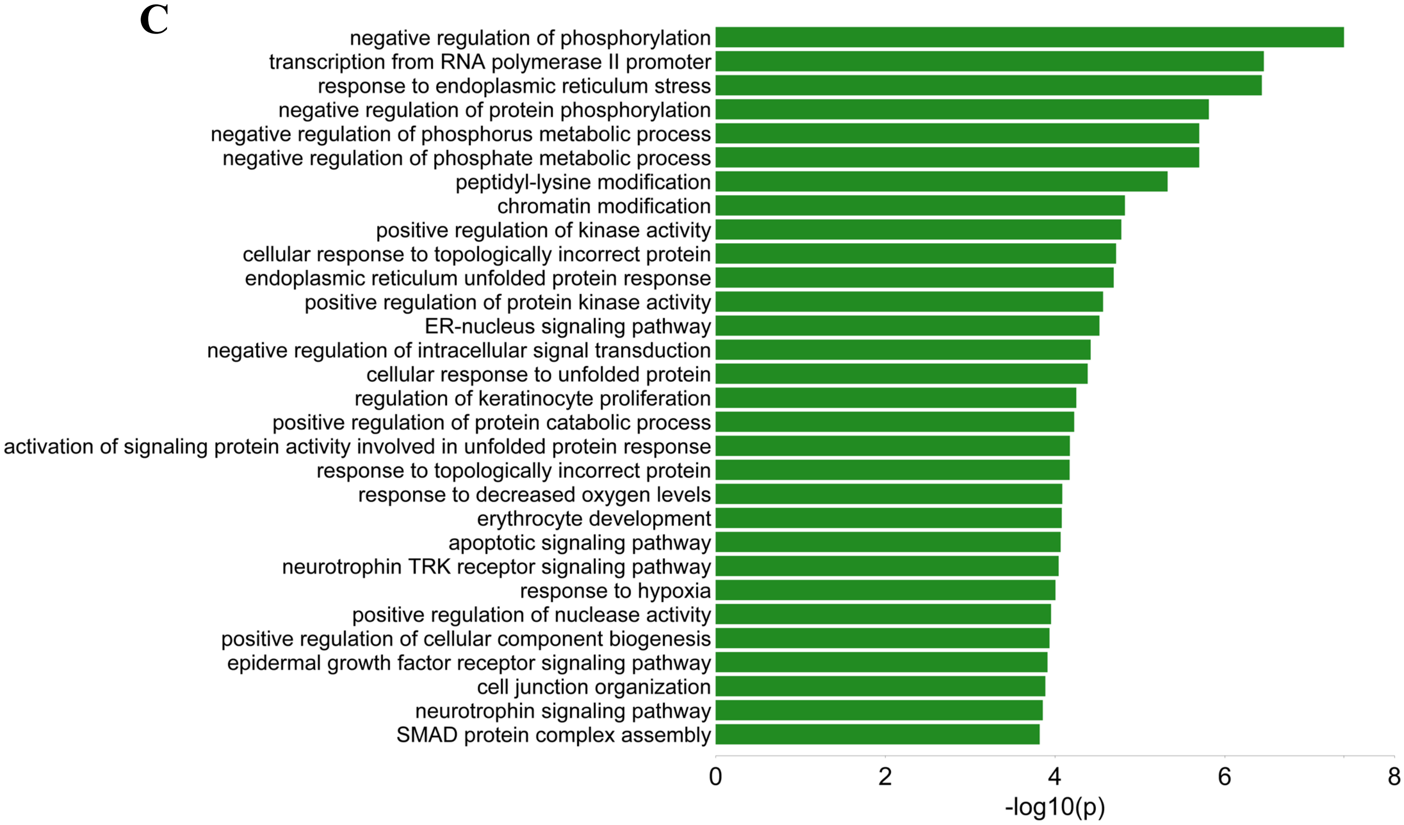
negative regulation of phosphorylation
transcription from RNA polymerase II promoter
response to endoplasmic reticulum stress
negative regulation of protein phosphorylation
negative regulation of phosphorus metabolic process
negative regulation of phosphate metabolic process
peptidyl-lysine modification
chromatin modification
positive regulation of kinase activity
cellular response to topologically incorrect protein
endoplasmic reticulum unfolded protein response
positive regulation of protein kinase activity
ER-nucleus signaling pathway
negative regulation of intracellular signal transduction
cellular response to unfolded protein
regulation of keratinocyte proliferation
positive regulation of protein catabolic process
activation of signaling protein activity involved in unfolded protein response
response to topologically incorrect protein
response to decreased oxygen levels
erythrocyte development
apoptotic signaling pathway
neurotrophin TRK receptor signaling pathway
response to hypoxia
positive regulation of nuclease activity
positive regulation of cellular component biogenesis
epidermal growth factor receptor signaling pathway
cell junction organization
neurotrophin signaling pathway
SMAD protein complex assembly
0
2
4
6
8
-log10(p)

D

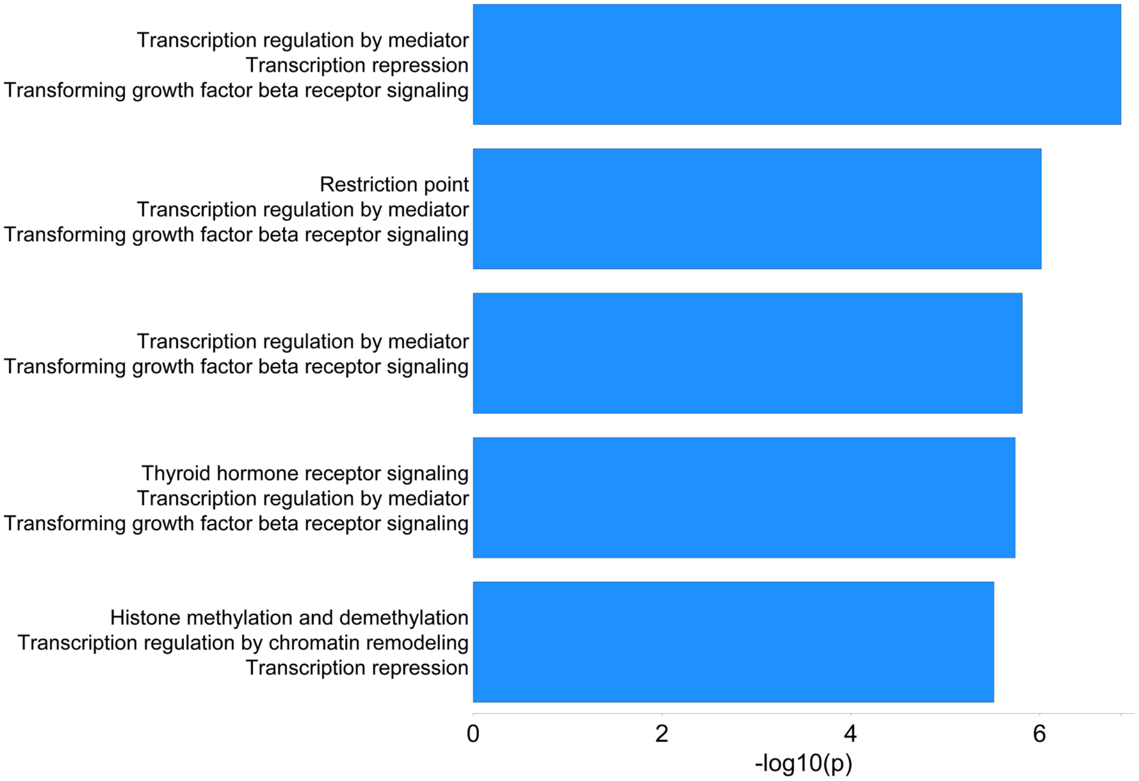
Transcription regulation by mediator
Transcription repression
Transforming growth factor beta receptor signaling
Restriction point
Transcription regulation by mediator
Transforming growth factor beta receptor signaling
Transcription regulation by mediator
Transforming growth factor beta receptor signaling
Thyroid hormone receptor signaling
Transcription regulation by mediator
Transforming growth factor beta receptor signaling
Histone methylation and demethylation
Transcription regulation by chromatin remodeling
Transcription repression
0
2
4
6
-log10(p)

E

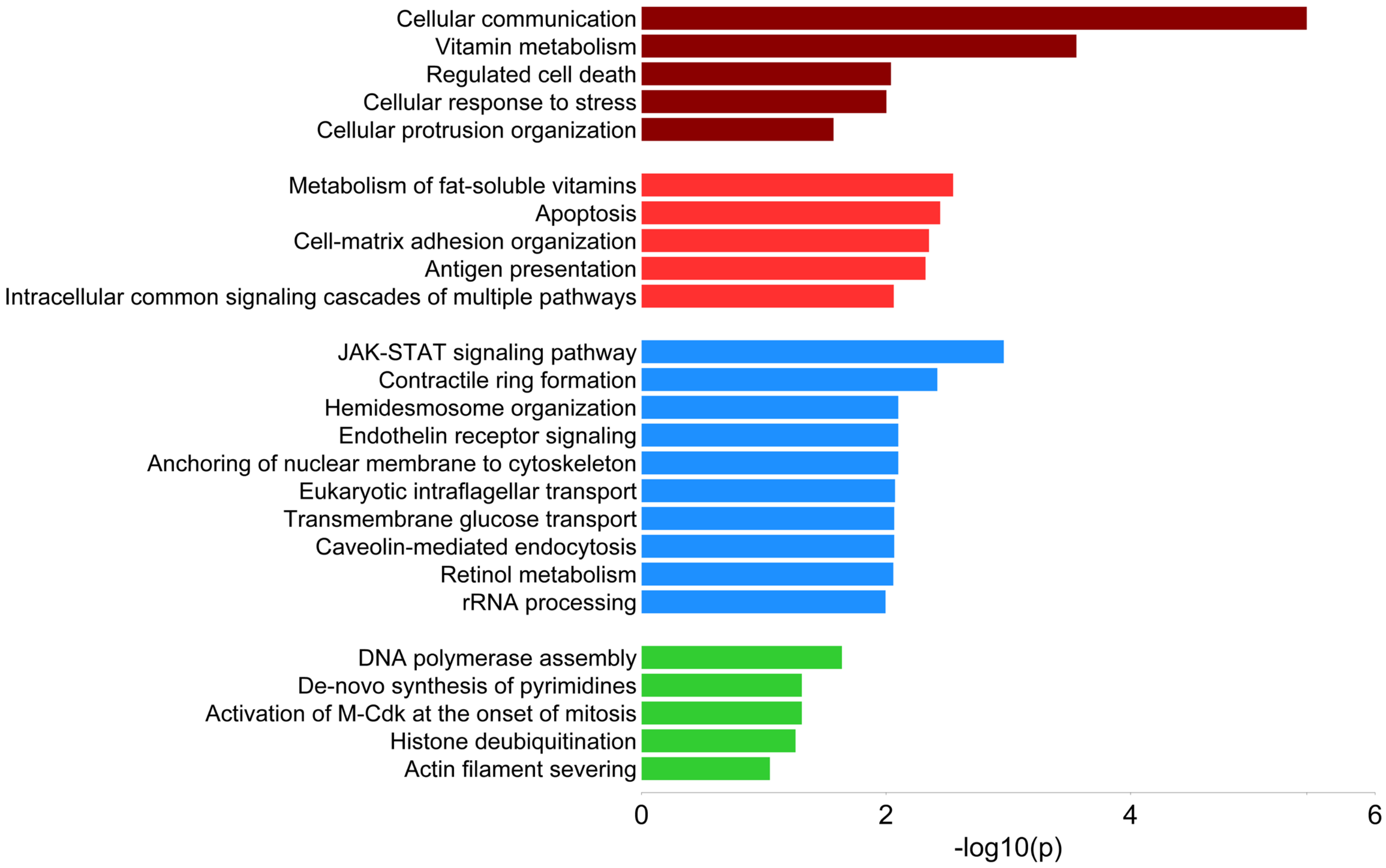

Cellular communication
Vitamin metabolism
Regulated cell death
Cellular response to stress
Cellular protrusion organization
Metabolism of fat-soluble vitamins
Apoptosis
Cell-matrix adhesion organization
Antigen presentation
Intracellular common signaling cascades of multiple pathways
JAK-STAT signaling pathway
Contractile ring formation
Hemidesmosome organization
Endothelin receptor signaling
Anchoring of nuclear membrane to cytoskeleton
Eukaryotic intraflagellar transport
Transmembrane glucose transport
Caveolin-mediated endocytosis
Retinol metabolism
rRNA processing
DNA polymerase assembly
De-novo synthesis of pyrimidines
Activation of M-Cdk at the onset of mitosis
Histone deubiquitination
Actin filament severing
0
2
4
6
-log10(p)

F

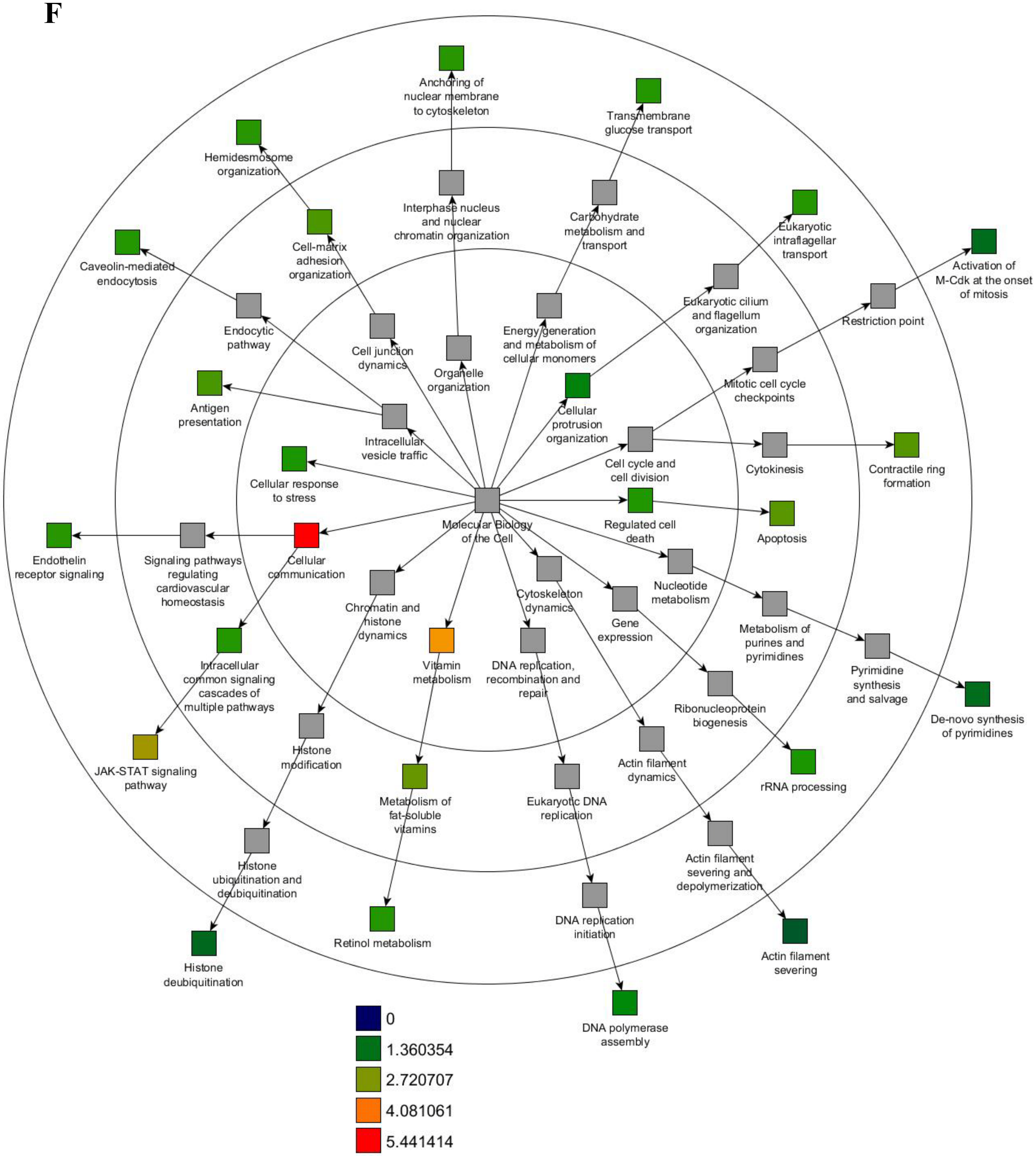
Anchoring of nuclear membrane to cytoskeleton
Transmembrane glucose transport
Hemidesmosome organization
Interphase nucleus and nuclear chromatin organization
Carbohydrate metabolism and transport
Eukaryotic intraflagellar transport
Cell-matrix adhesion organization
Caveolin-mediated endocytosis
Activation of M-Cdk at the onset of mitosis
Eukaryotic cilium and flagellum organization
Restriction point
Endocytic pathway
Cell junction dynamics
Energy generation and metabolism of cellular monomers
Organelle organization
Mitotic cell cycle checkpoints
Antigen presentation
Cellular protrusion organization
Intracellular vesicle traffic
Cell cycle and cell division
Cytokinesis
Contractile ring formation
Cellular response to stress
Regulated cell death
Apoptosis
Molecular Biology of the Cell
Endothelin receptor signaling
Signaling pathways regulating cardiovascular homeostasis
Cellular communication
Nucleotide metabolism
Chromatin and histone dynamics
Cytoskeleton dynamics
Gene expression
Metabolism of purines and pyrimidines
Intracellular common signaling cascades of multiple pathways
Vitamin metabolism
DNA replication, recombination and repair
Pyrimidine synthesis and salvage
Ribonucleoprotein biogenesis
De-novo synthesis of pyrimidines
JAK-STAT signaling pathway
Histone modification
Actin filament dynamics
rRNA processing
Metabolism of fat-soluble vitamins
Eukaryotic DNA replication
Histone ubiquitination and deubiquitination
Actin filament severing and depolymerization
Retinol metabolism
DNA replication initiation
Actin filament severing
Histone deubiquitination
DNA polymerase assembly
0
1.360354
2.720707
4.081061
5.441414

G

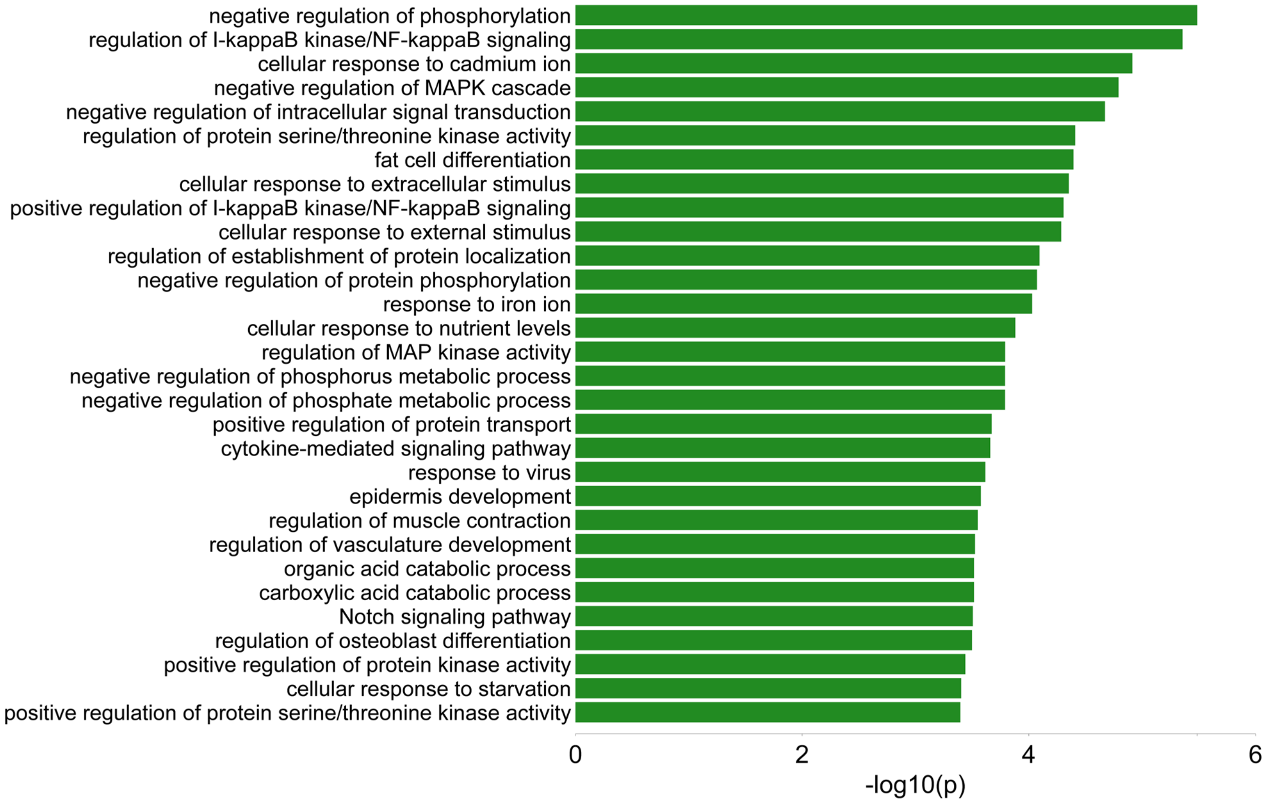

negative regulation of phosphorylation
regulation of I-kappaB kinase/NF-kappaB signaling
cellular response to cadmium ion
negative regulation of MAPK cascade
negative regulation of intracellular signal transduction
regulation of protein serine/threonine kinase activity
fat cell differentiation
cellular response to extracellular stimulus
positive regulation of I-kappaB kinase/NF-kappaB signaling
cellular response to external stimulus
regulation of establishment of protein localization
negative regulation of protein phosphorylation
response to iron ion
cellular response to nutrient levels
regulation of MAP kinase activity
negative regulation of phosphorus metabolic process
negative regulation of phosphate metabolic process
positive regulation of protein transport
cytokine-mediated signaling pathway
response to virus
epidermis development
regulation of muscle contraction
regulation of vasculature development
organic acid catabolic process
carboxylic acid catabolic process
Notch signaling pathway
regulation of osteoblast differentiation
positive regulation of protein kinase activity
cellular response to starvation
positive regulation of protein serine/threonine kinase activity
0
2
4
6
-log10(p)

H

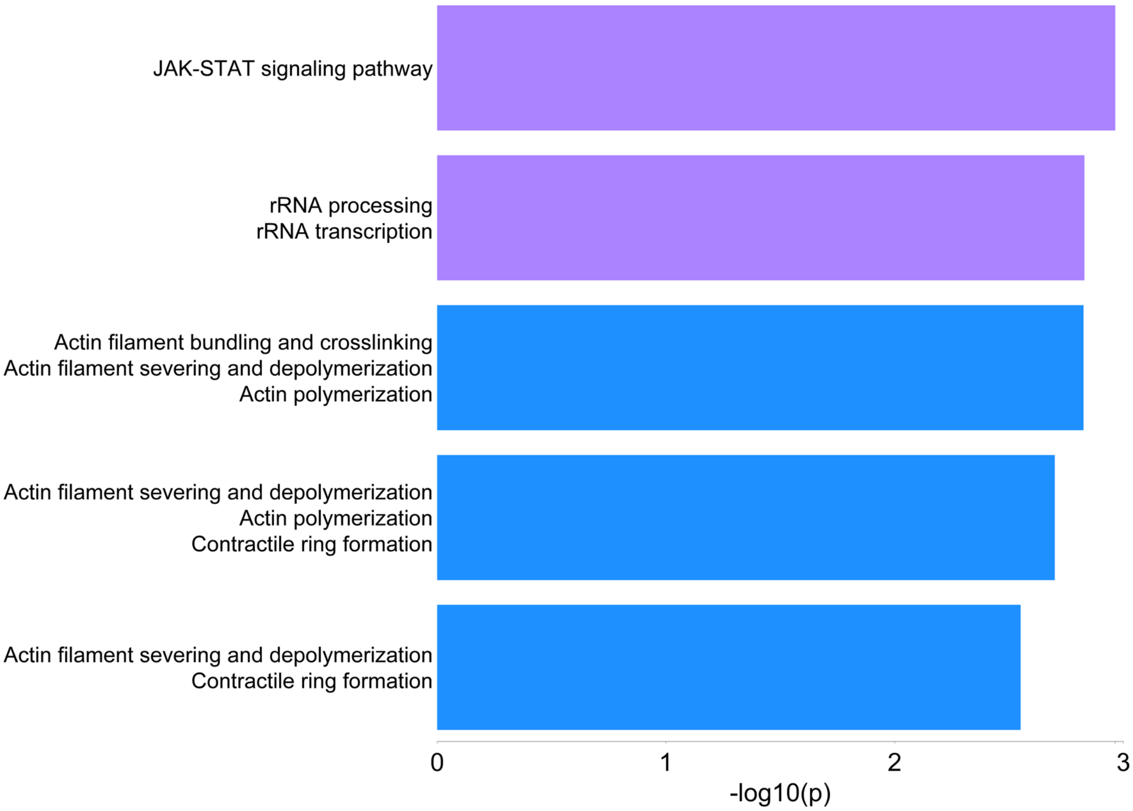

JAK-STAT signaling pathway
rRNA processing
rRNA transcription
Actin filament bundling and crosslinking
Actin filament severing and depolymerization
Actin polymerization
Actin filament severing and depolymerization
Actin polymerization
Contractile ring formation
Actin filament severing and depolymerization
Contractile ring formation
0
1
2
3
-log10(p)

I

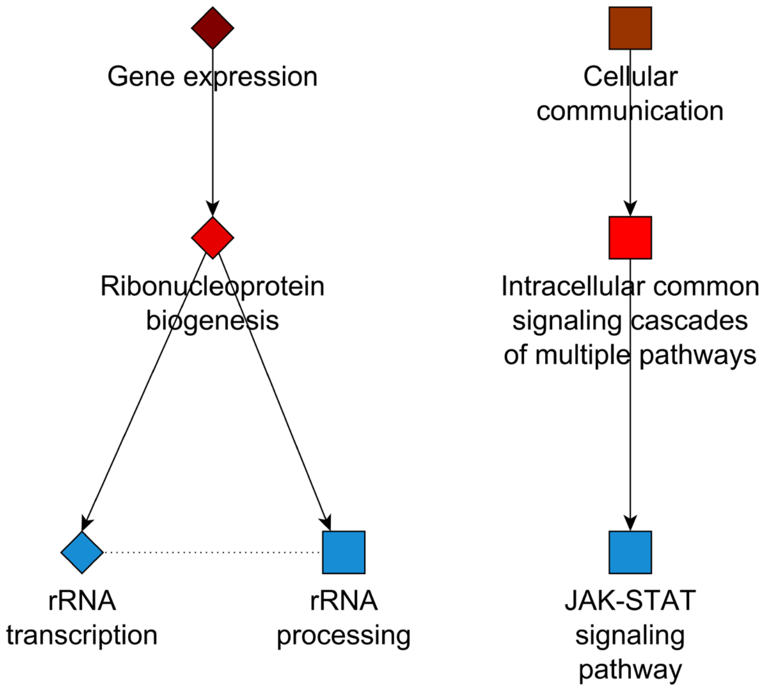

Supplementary Fig. S23: SCPs identified by standard and dynamic enrichment analysis of genes that were differentially expressed after erlotinib treatment. **(A-D) 6h erlotinib treatment (A)** Genes were subjected to standard enrichment analysis via Fisher's Exact test. Shown are the top 5 level-1, level-2 and level-4 as well as the top 10 level-3 SCPs that were predicted to be regulated by the identified protein interaction partners. Bars indicate minus $\log_{10}$(p-values). **(B)** Annotated parent-child relationships between the predicted SCP. Colors indicate minus $\log_{10}$(p-values). SCPs in gray were not among the top predicted SCPs but are descendents of SCPs that were among the top predictions. **(C)** Top 30 predicted Gene Ontology biological processes that were identified based on standard enrichment analysis.**(D)** Top 5 predicted SCPs or SCP units that were identified via dynamic enrichment analysis. See Fig. 5 for details. Blue bars: minus $\log_{10}$ (p-values) of those single SCPs or SCP-units that are part of the largest SCP networks shown in Fig. 5. **(E-I) 24h erlotinib treatment (E)** Genes were subjected to standard enrichment analysis via Fisher's Exact test. Shown are the top 5 level-1, level-2 and level-4 as well as the top 10 level-3 SCPs that are predicted to be regulated by the identified protein interaction partners. Bars indicate minus $\log_{10}$(p-values). **(F)** Annotated parent-child relationships between the predicted SCP. Colors indicate minus $\log_{10}$(p-values). SCPs in gray were not among the top predicted SCPs but are descendents of SCPs that were among the top predictions. **(G)** Top 30 predicted Gene Ontology biological processes that were identified based on standard enrichment analysis. **(H)** Top 5 predicted SCPs or SCP units that were identified via dynamic enrichment analysis. See Fig. 5 for details. Blue bars: minus $\log_{10}$ (p-values) of those single SCPs or SCP-units that are part of the largest SCP networks shown in Fig. 5. Purple bars: minus $\log_{10}$ (p-values) of all other predictions. **(I)** Predicted level-3 SCPs (light blue) that were not part of the largest SCP network (violet bar in Supplementary Fig. S21D) and their annotated level-1 grandparent and level-2 parent SCPs.

Description of attached files

Supplementary Table S1: Hierarchical structure of MBC Ontology (A) MBC Ontology SCPs, PubMed queries and references. (B) OBO-format of the hierarchy of the MBC Ontology.

Supplementary Table S2: Organization of the different databases and dictionaries that were generated to populate the MBC Ontology. Downloaded databases were published databases that we used to generate our own databases and dictionaries. In some cases we first generated (sub-)databases that were merged to the final dictionary (e.g. we first generated 'Protein complexes generated based on CORUM database' from the CORUM database before we added this database to the gene group dictionary). Columns indicate the name of the columns in the corresponding database or dictionary. The entries that were found in the column of a database on the left side were added to the columns in the same rows of the databases shown on the middle or right site. For example, the entries in the columns 'Synonym' of the NCBI gene info database, 'Alias symbol' of the HGNC database and 'Synonym' of our manually generated database were added as 'Synonyms' in the Gene dictionary. Similarly, the entries in the column 'Complex name' of the CORUM complex database were added as 'Description' to the 'Protein complexes generated based on CORUM database'.

In general, our own database contains a column 'description' as well as a column 'description aliases' that contain the full name and alternative full names of the biological object, a column 'abbreviation' or 'symbol' as well as a column 'abbreviation aliases' or 'synonym' that contain the abbreviation and alternative abbreviations for the biological object.

Supplementary Table S3: Gene description aliases and synonyms that were identified manually. For glossary see Supplementary Table S2.

Supplementary Table S4: Proposed gene synonyms identified as terms in brackets. The table shows the labels of the different label sets that were used to identify complexes and families by text mining of the NCBI descriptions. If an identified label is (directly) followed by one of the gene groups exclusion words, it won't be considered any more. For glossary see Supplementary Table S2.

Supplementary Table S5: Gene dictionary. For glossary see Supplementary Table S2.

Supplementary Table S6: Expressions used for the identification of gene groups by text mining. For glossary see Supplementary Table S2.

Supplementary Table S7: Manual replacements for gene groups generated by textmining. For glossary see Supplementary Table S2.

Supplementary Table S8: Gene groups generated by text mining of gene descriptions. For glossary see Supplementary Table S2.

Supplementary Table S9: CORUM abbreviations and description aliases that were identified manually. For glossary see Supplementary Table S2.

Supplementary Table S10: Protein complexes generated based on CORUM database. For glossary see Supplementary Table S2.

Supplementary Table S11: HGNC abbreviations that were identified manually. For glossary see Supplementary Table S2.

Supplementary Table S12: Gene groups generated based on HGNC database. For glossary see Supplementary Table S2.

Supplementary Table S13: Gene groups that were identified manually. For glossary see Supplementary Table S2.

Supplementary Table S14: Gene groups that were deleted from gene group dictionary. For glossary see Supplementary Table S2.

Supplementary Table S15: Gene group dictionary. For glossary see Supplementary Table S2.

Supplementary Table S16: Description aliases for metabolites and complex lipids and sugars that were identified manually. For glossary see Supplementary Table S2.

Supplementary Table S17: Metabolites and complex lipids and sugars that were identified manually. For glossary see Supplementary Table S2.

Supplementary Table S18: Metabolite and complex lipid and sugar dictionary. For glossary see Supplementary Table S2.

Supplementary Table S19: Protein domains that were identified manually. For glossary see Supplementary Table S2.

Supplementary Table S20: Protein domain dictionary. For glossary see Supplementary Table S2.

Supplementary Table S21: Diseases that were identified manually. For glossary see Supplementary Table S2.

Supplementary Table S22: Disease dictionary. For glossary see Supplementary Table S2.

Supplementary Table S23: Drugs that were identified manually. For glossary see Supplementary Table S2.

Supplementary Table S24: Drug dictionary. For glossary see Supplementary Table S2.

Supplementary Table S25: Sub-cellular structures that were identified manually. For glossary see Supplementary Table S2.

Supplementary Table S26: Sub-cellular processes that were identified manually. For glossary see Supplementary Table S2.

Supplementary Table S27: Sub-cellular process dictionary. For glossary see Supplementary Table S2.

Supplementary Table S28: Confounding terms that were identified manually. For glossary see Supplementary Table S2.

Supplementary Table S29: Background terms. For glossary see Supplementary Table S2.

Supplementary Table S30: Expressions that are considered during the text mining approach. If an article contains an article exclusion word, it won't be considered any more. Any key terms that are directly followed by a key term exclusion word (i.e. without any intermittent word), will not be considered. For glossary see Supplementary Table S2.

Supplementary Table S31: Overall dictionary. For glossary see Supplementary Table S2.

Supplementary Table S32: MBC ontology - gene SCP associations.

Supplementary Table S33: Overlap of genes between parent and children SCPs.

Supplementary Table S34: Overlap of genes between parent and children SCPs after removal of children SCPs.

Supplementary Table S35: Inferred interactions between level-3 SCP.

Supplementary Table S36: Standard and dynamic enrichment analysis of case studies.

Supplementary Text 1: Manual validation approach for gene-SCP associations. For each gene we selected up to 5 example sentences from the abstracts of the SCP-specific abstract set and printed them into the text file. Additionally, we added the gene summary of that particular gene that was down loaded from the NCBI website. T: True positive, F: False positive, M: False positive that arose from the misinterpretation of a non-gene term as a gene, S: False positive gene that belongs to a sibling process

Suppl. References (Supplementary Table S1A):

1. Alford, A.I. & Hankenson, K.D. Matricellular proteins: Extracellular modulators of bone development, remodeling, and regeneration. *Bone*38, 749-757 (2006).
2. Bornstein, P. & Sage, E.H. Matricellular proteins: extracellular modulators of cell function. *Current opinion in cell biology***14**, 608-616 (2002).
3. Theocharis, A.D., Skandalis, S.S., Gialeli, C. & Karamanos, N.K. Extracellular matrix structure. *Advanced drug delivery reviews***97**, 4-27 (2016).
4. English, A.R. & Voeltz, G.K. Endoplasmic reticulum structure and interconnections with other organelles. *Cold Spring Harbor perspectives in biology***5**, a013227 (2013).
5. Goyal, U. & Blackstone, C. Untangling the web: mechanisms underlying ER network formation. *Biochimica et biophysica acta***1833**, 2492-2498 (2013).
6. Mekhail, K. & Moazed, D. The nuclear envelope in genome organization, expression and stability. *Nature reviews. Molecular cell biology***11**, 317-328 (2010).
7. Simon, J.A. & Kingston, R.E. Mechanisms of polycomb gene silencing: knowns and unknowns. *Nature reviews. Molecular cell biology***10**, 697-708 (2009).
8. Van Laar, V.S. & Berman, S.B. The interplay of neuronal mitochondrial dynamics and bioenergetics: implications for Parkinson's disease. *Neurobiology of disease***51**, 43-55 (2013).
9. Conduit, P.T., Wainman, A. & Raff, J.W. Centrosome function and assembly in animal cells. *Nature reviews. Molecular cell biology***16**, 611-624 (2015).
10. Green, R.A., Paluch, E. & Oegema, K. Cytokinesis in animal cells. *Annual review of cell and developmental biology***28**, 29-58 (2012).
11. Morrison, A.J. & Shen, X. Chromatin remodelling beyond transcription: the INO80 and SWR1 complexes. *Nature reviews. Molecular cell biology***10**, 373-384 (2009).
12. Ceccaldi, R., Rondinelli, B. & D'Andrea, A.D. Repair Pathway Choices and Consequences at the Double-Strand Break. *Trends in cell biology***26**, 52-64 (2016).
13. Duxin, J.P. & Walter, J.C. What is the DNA repair defect underlying Fanconi anemia? *Current opinion in cell biology***37**, 49-60 (2015).
14. Anitei, M. et al. A high-throughput siRNA screen identifies genes that regulate mannose 6-phosphate receptor trafficking. *Journal of cell science***127**, 5079-5092 (2014).
15. Hashemi, H.F. & Goodman, J.M. The life cycle of lipid droplets. *Current opinion in cell biology***33**, 119-124 (2015).
16. Porrua, O. & Libri, D. Transcription termination and the control of the transcriptome: why, where and how to stop. *Nature reviews. Molecular cell biology***16**, 190-202 (2015).
17. Allen, B.L. & Taatjes, D.J. The Mediator complex: a central integrator of transcription. *Nature reviews. Molecular cell biology***16**, 155-166 (2015).
18. Sainsbury, S., Bernecky, C. & Cramer, P. Structural basis of transcription initiation by RNA polymerase II. *Nature reviews. Molecular cell biology***16**, 129-143 (2015).
19. Matera, A.G. & Wang, Z. A day in the life of the spliceosome. *Nature reviews. Molecular cell biology***15**, 108-121 (2014).
20. Greve, T.S., Judson, R.L. & Blelloch, R. microRNA control of mouse and human pluripotent stem cell behavior. *Annual review of cell and developmental biology***29**, 213-239 (2013).
21. Houseley, J. & Tollervey, D. The many pathways of RNA degradation. *Cell***136**, 763-776 (2009).
22. Oeffinger, M. & Montpetit, B. Emerging properties of nuclear RNP biogenesis and export. *Current opinion in cell biology***34**, 46-53 (2015).
23. McIlwain, D.R., Berger, T. & Mak, T.W. Caspase functions in cell death and disease. *Cold Spring Harbor perspectives in biology***5**, a008656 (2013).
24. Humphrey, J.D., Dufresne, E.R. & Schwartz, M.A. Mechanotransduction and extracellular matrix homeostasis. *Nature reviews. Molecular cell biology***15**, 802-812 (2014).
25. Mouw, J.K., Ou, G. & Weaver, V.M. Extracellular matrix assembly: a multiscale deconstruction. *Nature reviews. Molecular cell biology***15**, 771-785 (2014).

26. Bonnans, C., Chou, J. & Werb, Z. Remodelling the extracellular matrix in development and disease. *Nature reviews. Molecular cell biology***15**, 786-801 (2014).
27. Papke, C.L. & Yanagisawa, H. Fibulin-4 and fibulin-5 in elastogenesis and beyond: Insights from mouse and human studies. *Matrix biology : journal of the International Society for Matrix Biology***37**, 142-149 (2014).
28. Yan, N. & Shi, Y. Mechanisms of apoptosis through structural biology. *Annual review of cell and developmental biology***21**, 35-56 (2005).
29. Yuan, S. & Akey, C.W. Apoptosome structure, assembly, and procaspase activation. *Structure***21**, 501-515 (2013).
30. Adeva, M.M., Souto, G., Blanco, N. & Donapetry, C. Ammonium metabolism in humans. *Metabolism: clinical and experimental***61**, 1495-1511 (2012).
31. Locasale, J.W. Serine, glycine and one-carbon units: cancer metabolism in full circle. *Nature reviews. Cancer***13**, 572-583 (2013).
32. Hettmer, S., McCarter, R., Ladisch, S. & Kaucic, K. Alterations in neuroblastoma ganglioside synthesis by induction of GD1b synthase by retinoic acid. *British journal of cancer***91**, 389-397 (2004).
33. Don, A.S., Lim, X.Y. & Couttas, T.A. Re-configuration of sphingolipid metabolism by oncogenic transformation. *Biomolecules***4**, 315-353 (2014).
34. De Matteis, M.A. & Rega, L.R. Endoplasmic reticulum-Golgi complex membrane contact sites. *Current opinion in cell biology***35**, 43-50 (2015).
35. Du, X., Brown, A.J. & Yang, H. Novel mechanisms of intracellular cholesterol transport: oxysterol-binding proteins and membrane contact sites. *Current opinion in cell biology***35**, 37-42 (2015).
36. Hines, R.N. & McCarver, D.G. The ontogeny of human drug-metabolizing enzymes: phase I oxidative enzymes. *The Journal of pharmacology and experimental therapeutics***300**, 355-360 (2002).
37. Chandra, P. & Brouwer, K.L. The complexities of hepatic drug transport: current knowledge and emerging concepts. *Pharmaceutical research***21**, 719-735 (2004).
